# Olfactory Stimuli Increase Presence in Virtual Environments

**DOI:** 10.1371/journal.pone.0157568

**Published:** 2016-06-16

**Authors:** Benson G. Munyan, Sandra M. Neer, Deborah C. Beidel, Florian Jentsch

**Affiliations:** Department of Psychology, University of Central Florida, Orlando, Florida, United States of America; Universidade de São Paulo, BRAZIL

## Abstract

**Background:**

Exposure therapy (EXP) is the most empirically supported treatment for anxiety and trauma-related disorders. EXP consists of repeated exposure to a feared object or situation in the absence of the feared outcome in order to extinguish associated anxiety. Key to the success of EXP is the need to present the feared object/event/situation in as much detail and utilizing as many sensory modalities as possible, in order to augment the sense of presence during exposure sessions. Various technologies used to augment the exposure therapy process by presenting multi-sensory cues (e.g., sights, smells, sounds). Studies have shown that scents can elicit emotionally charged memories, but no prior research has examined the effect of olfactory stimuli upon the patient’s sense of presence during simulated exposure tasks.

**Methods:**

60 adult participants navigated a mildly anxiety-producing virtual environment (VE) similar to those used in the treatment of anxiety disorders. Participants had no autobiographical memory associated with the VE. State anxiety, Presence ratings, and electrodermal (EDA) activity were collected throughout the experiment.

**Results:**

Utilizing a Bonferroni corrected Linear Mixed Model, our results showed statistically significant relationships between olfactory stimuli and presence as assessed by both the Igroup Presence Questionnaire (IPQ: *R*^2^ = 0.85, (*F*(3,52) = 6.625, *p* = 0.0007) and a single item visual-analogue scale (*R*^2^ = 0.85, (*F*(3,52) = 5.382, *p* = 0.0027). State anxiety was unaffected by the presence or absence of olfactory cues. EDA was unaffected by experimental condition.

**Conclusion:**

Olfactory stimuli increase presence in virtual environments that approximate those typical in exposure therapy, but did not increase EDA. Additionally, once administered, the removal of scents resulted in a disproportionate decrease in presence. Implications for incorporating the use of scents to increase the efficacy of exposure therapy is discussed.

## Introduction

### Anxiety Disorders

Anxiety disorders share features of excessive fear, worry, and related behavioral disturbances [1] and are among the most common mental health problems seen in the medical community today. Estimates suggest that 19.5% - 28.8% of people within the United States have at least one anxiety disorder [2] with lifetime prevalence estimates of 12.1% and 12.5%, for social phobia and specific phobia, respectively. Mean age of onset for anxiety disorders is 11 years old, which is earlier than age of onset of substance disorders (age 20) and mood disorders (age 30) [3]. As such, anxiety disorders begin consuming resources far earlier than other types of mental disorders. The direct financial costs of anxiety disorders may take the form of counseling, hospitalization, and medications [4]. Indirect financial costs may include reduced productivity and absenteeism from work [5]. Direct and indirect costs combined, Greenberg estimated that anxiety disorders cost nearly $42.3 billion dollars during the 1990’s (after adjusting for inflation, $75 billion in 2013 dollars). In addition to financial burdens, Greenberg and colleagues [4]specified impaired social functioning, increased likelihood of dropping out of school, teenage pregnancy, marital instability, poor career choices, and required caretaking by family and friends as costs associated with anxiety disorders. Anxiety disorders are also associated with increased substance abuse and dependence which likely increase direct and indirect costs [6].

### Trauma and Stress-related disorders

Trauma- and stressor-related disorders are those in which exposure to a traumatic or very stressful event is explicitly included in the diagnostic criteria. This DSM-5 category includes posttraumatic stress disorder (PTSD) and acute stress disorder. Common types of traumatic events include assaultive violence, injury or shocking experiences, and even learning about trauma to others [7]. Trauma- and stressor-related disorders are closely related to anxiety disorders and until the publication of DSM-5 fell under the diagnostic umbrella of anxiety disorders [1]. Whereas individuals with anxiety disorders often exhibit anxiety or fear-based symptoms, those with disorders associated with stress and trauma most often display anhedonic and dysphoric symptoms, externalized anger and aggressive symptoms, or dissociative symptoms in addition to anxiety and fear-related symptoms [1].

One common symptom shared by anxiety and trauma-related disorders is behavioral avoidance. By preventing memories of the traumatic event from surfacing, those engaging in avoidant behavior can also prevent the negative and fearful thoughts and feelings associated with the traumatic memory thus protecting themselves from perceived danger and further harm. However, by avoiding those same thoughts and feelings, they prevent themselves from learning new and more appropriate response patterns [8]. Ehlers and Clark [9] describe avoidance as a maladaptive control strategy that short circuits disconfirmation of negative appraisals, which result in the maintenance of perceived current threat. This type of behavior has been documented in various populations with PTSD, including combat veterans [10], victims of sexual assault [11], and motor vehicle accident victims [12]. However, avoidance is also seen in many anxiety disorders, including social anxiety disorder, specific phobia, panic disorder, separation anxiety disorder, and agoraphobia [1]. Preventing avoidant behavior and encouraging patients to face anxiety-provoking situations can correct incompatible and erroneous information with more appropriate behavioral responses that enable better daily functioning.

### Exposure therapy

Exposure Therapy (EXP) has been shown to be effective in the treatment of anxiety and trauma-related disorders [13]. EXP is analogous in humans to fear extinction models used in animals [14] and is based upon the principles of classical conditioning discovered by Pavlov [15] and later explored by Watson and Rayner [16]. An example of this might be conditioned taste aversion [17], where after eating a favorite food, the individual becomes severely ill and afterwards no longer desires the food that preceded becoming ill. With respect to anxiety disorders, an example of classical conditioning in PTSD might include avoidance of driving after coming into contact with a roadside bomb that detonated, threatening the life of the driver and/or passengers. In specific phobia, a child might develop an extreme fear response to dogs after being chased or bitten and subsequently avoids leaving the house due to fear of encountering a dog. Exposure therapy seeks to extinguish learned behaviors that are or have become maladaptive by exposing patients to the anxiety or fear-producing stimulus (or a facsimile of that stimulus) without exposing them to the danger, thus allowing new information and expectations to be learned.

EXP is a highly researched and effective treatment for anxiety disorders [18]. EXP has been included in several versions of cognitive behavioral therapy (CBT) that have proven to be effective for numerous different populations, including those who have been in motor vehicular accidents [19] and victims of sexual assault [20]. There is also a wide body of literature supporting the effectiveness of EXP in treating PTSD [18, 21]. More recent treatments have incorporated virtual reality (VR) equipment and have been shown to be effective in populations that survived terrorist attacks [22] and those with combat-related PTSD [23]. There are a number of important benefits of using VR in EXP; it is possible to expose patients to a greater number of situations and stimuli without leaving the therapists office, exposure stimuli can be precisely controlled, decreased time and expense formulating exposure sessions, and exposure with VR poses less risk of harm or embarrassment [24]. Additionally, Wiederhold, Jang, Gevirtz, Kim, Kim and Wiederhold [25] found that exposure therapy that included VR was more effective than imaginal exposure therapy in the treatment of fear of flying. VR was also shown to be at least as effective as in vivo in the treatment of acrophobia [26].

The core components of exposure therapy include a) imagining the traumatic event, recanting the experience, and reprocessing the memory, and/or b) in-vivo exposure, in which situations and objects that may be associated with the trauma are confronted. In imaginal exposure the patient is asked to visualize the trauma as vividly as possible while the therapist provides information about all of the senses to increase an individual’s ability to imagine the trauma. By adding actual sights, sounds, and smells, the individual may be better able to imagine the scene.

### Olfaction Overview

Olfaction, or the ability to smell, is the result of responses by receptor cells to chemical stimuli. This system is called chemosensory, and is found in nearly all animal species [27]. Odor perception begins with the olfactory epithelium (OE), a small area of specialized tissue located inside the nasal cavity. The OE is directly responsible for the detection of the volatile chemical compounds that comprise scents. From the OE, information is passed to the olfactory bulb and eventually is delivered to the amygdala, thalamus, hippocampus, and hypothalamus.

It has been suggested that the amygdala is activated based on a combination of the valence and intensity properties of an odor [28]. It is widely accepted that the hippocampus plays an important role in the formation of new memories about experienced events [29]. Specifically, the hippocampus is linked to the ability to navigate an environment and recall the events that occur there [29], which becomes important when navigating a virtual environment. It has also been suggested that the amygdala and hippocampus act in unison when emotion and memory are connected. Phelps [30] described the amygdala’s ability to modulate the encoding and storage of hippocampal-dependent memories in addition to the hippocampus’ influence on amygdala responses when emotional stimuli, such as those encountered during traumatic events, are presented.

It has long been suggested that smells are the best reminders of past experiences, a piece of folk wisdom first described in Swann’s Way [31]. In fact, research has shown olfactory stimuli to result in more emotionally potent memory recall than verbal and visual modalities [32, 33]. Olfactory stimuli have been utilized in exposure therapy with combat veterans to augment the sense of environment, and have included scents such as burning rubber, cordite, garbage, body odor, gunpowder, and diesel fuel [23]. Kline and Rausch [34] described the impact of olfactory stimuli as precipitants of flashbacks in Vietnam veterans. Vermetten and Bremner [35] documented a particularly vivid example of the emotional impact olfactory stimuli can have when paired to traumatic events:

*This morning*, *I noticed local firefighting equipment on the road just past my home*. *The fire police let me pass since our house is on the corner*. *Arriving home*, *I found my wife out on the back deck watching a fire that was about 300 feet away*. *This is when I noticed the smell of burning rubber*, *together with a faint smell of fuel oil or diesel oil*. *My wife stated she was worried about me because I was standing on the deck as if I was daydreaming for some minutes without responding to her*. *The smell brought to my mind the image of this burning Amtrak*, *again so vivid*. *The Amtrak was hit*. *The front door/ramp was open; both crew hatches were open and pouring out smoke and flame*. *Thick*, *black*, *acid smoke was boiling out of the troop compartment*. *There was an overpowering smell of burning rubber*. *I remember that smell and what it looked like that day vividly*. *There was nothing I could have done to save the people in the Amtrak*. *Fifteen Marines and 3 crewmembers died there that day*. *I felt the same hopelessness as I felt that day*. *I felt bad in my stomach*, *got a headache*, *and had a feeling of futility or finality when I thought about that incident*. (Page 203, paragraph 3).

Despite what appears to be general acceptance of the link between memory and olfaction, no identifiable research has focused on the role of olfaction in the treatment of anxiety disorders in general. Olfactory stimuli have been shown to increase presence in general virtual environments [36], but no research could be identified that sought to quantify olfaction’s effect specifically with respect to simulated exposure tasks like those used in the treatment of anxiety and trauma-related disorders. If olfactory stimuli enhance the sense of presence in an environment during simulated exposure tasks, it seems logical that exposure therapy may be more effective when olfactory cues are added.

### Presence

Presence, when used to describe immersive feelings in virtual reality (VR), has been conceptualized and defined in different ways (for a review, see Lombard and Ditton [37]). Presence is often described from the concept of transportation [38], that is to say, people are usually considered “present” when they feel as if they are actually in the virtual world. Creative methods utilized for the purposes of increasing presence in virtual reality may include the use of tactile feedback, surround or 3D sound, and head mounted displays (HMDs) with high visual fidelity.

One benefit of elevated levels of presence is that when asked to recall, users remember the environment as if it was a real place instead of a simulated location [39]. Similarly, VR environments may produce the same emotions and physical reactions as their real-world counterparts when the level of presence experienced by the user is sufficiently high [37]. The ability to evoke similar emotions and physical reactions is particularly useful for clinical applications. For example, Hodges, Kooper, Meyer, De Graaff, Rothbaum, Opdyke et al. [40] found that participants with clinical diagnoses of acrophobia reported increased anxiety when presented VR that included great heights. This ability to evoke real emotions from artificial environments has presumably led to the use of VR for the treatment of numerous anxiety disorders [41, 42], as well as PTSD [24, 43].

It is generally believed that the more senses are utilized by a medium, the greater its ability to generate a sense of presence [44]. In fact, many studies have examined the effect of screen size [45], sound, and multi-speaker systems [46] on presence. Tactile stimuli also increase presence [47], and have been used in the treatment of combat-related PTSD [23]. It has also been suggested that olfactory delivery systems be introduced to VR [48]. Given the substantial research supporting the relationship between olfaction and strong emotional memory [32, 49], it seems logical to explore the effect of olfaction on presence during virtual-reality assisted exposure therapy.

However, the utility of olfactory stimuli (OS) to increase presence during simulated exposure therapy tasks is not yet known. In this investigation, we examined whether OS were associated with changes in presence and state anxiety responses within a virtual environment. Specifically, we hypothesized that participants would experience higher levels of presence, greater electrodermal activity, and state anxiety when engaged in VE’s while receiving OS.

## Methods

### Consent

Oral consent was obtained by participants prior to participation as approved by the University of Central Florida (UCF) IRB (IRB Number: SBE-14-10650). The UCF IRB classified this human research as “minimal risk”, and directed the researchers to obtain oral consent rather than written consent. Oral consent was documented by video security systems within the research facility.

### Sample

The sample consisted of 60 participants between the ages of 18 and 31 years of age (*M* = 20.48, *SD* = 3.13). Sixty-five percent (65%) were male (*n* = 39), while ethnicity varied, including 38 Caucasians, 11 Hispanics, 6 African Americans, 2 Asians, and 3 who identified as Other (e.g., of mixed ethnic background). To be included in the study, participants were required to achieve a passing score on a brief test of olfactory function (see below). A history of seizures, epilepsy, or current prescriptions for beta-blocking or anxiety medications excluded individual participants from participating in the study. No participants had autobiographical memories consistent with the VE.

### Measures

#### Quick Smell Identification Test (QSIT)

The Quick Smell Identification Test (QSIT; Sensonics, Inc., Haddon Heights, NJ) is a three-item multiple-choice test consisting of three microencapsulated odorant strips. Jackman and Doty [50] found the Q-SIT to be highly reliable over time (*r* = 0.87) and highly sensitive to identifying olfactory loss, particularly in those with severe olfactory deficits. In addition, they found that a score of two on the QSIT provided sensitivity (true positive) and specificity (true negative) of 99% and 43%, respectively. Positive predictive power and negative predictive power were found to be 91% and 42%, respectively.

#### State-Trait Anxiety Inventory (STAI)

The state-trait anxiety inventory [51] is a 40-item, self-report measure designed to measure both the transient state of arousal subjectively experienced as anxiety and the more chronic emotional presence of anxiety. It has excellent psychometric properties [52] and has been adapted for use in over 40 languages. It has a 6^th^ grade reading level, can be administered individually or in groups, and has a response burden of approximately ten minutes. The STAI assesses items based on a four-factor structure, which is comprised of two primary factors: state anxiety and trait anxiety. Both state and trait anxiety are further comprised of two additional factors, Anxiety Absent and Anxiety Present. Items on the STAI range from “I am Calm” (State Anxiety, Anxiety Absent) to “I worry too much over something that doesn’t really matter (Trait Anxiety, Anxiety Present).

#### Igroup Presence Questionnaire (IPQ)

The Igroup Presence Questionnaire is a 14-item self-report questionnaire designed to measure presence utilizing a 7-point Likert scale [53] that loads onto three subscales; spatial presence (the sense of physically being in the virtual environment), involvement (focus on the VE and involvement experienced), and experienced realism (subjective realism of the VE). Items range from “How aware were you of the real world while navigating in the virtual world?” to “How real did the virtual world seem to you?”

#### Immersive Tendencies Questionnaire (ITQ)

The Immersive Tendencies Questionnaire [54] is a 29-item self-report measure designed to assess individual tendencies towards immersing in different mediums. The items in this questionnaire measure the participant’s involvement in many different daily activities, such as watching television, reading books, or enjoying movies. As involvement can result in more immersion, it is thought that those who become more involved will also have greater immersive tendencies.

#### Presence Visual-Analogue Scale (PVAS)

Participants were asked to rate their level of immersion during the experiment to determine presence on a visual-analogue scale (VAS). Visual-analogue scales have been demonstrated to accurately index anxiety [55]. It has been shown that VASs have moderate to strong correlations with Likert based items [56]. The VAS response will be converted to units of measurement (millimeters) for data analysis purposes. VASs have superior metrical characteristics than discrete scales and can have a wider range of statistical methods applied to their measurements [57].

#### Presence Rating Scale (PRS)

Participants were asked at evoked events to rate their current level of presence during the exposure task. This rating was on a 7-point Likert scale to remain consistent with the Likert scale of the IPQ. The question, “How present do you feel?” was anchored at one (not at all) and seven (very much).

#### Simulator Sickness Questionnaire (SSQ)

The Simulator Sickness Questionnaire was developed by Kennedy, Lane, Berbaum and Lilienthal [58]. It is a 16-item self-report scale used to rate common symptoms of simulator sickness on a 4-point scale. Such symptoms include general discomfort, headache, eyestrain, sweating, and vertigo. Information about the user’s present state of health was solicited prior to simulator use, as well as after simulator use. The SSQ was used for pre- and post-experimental assessment to assess symptoms commonly associated with VR use.

#### Electrodermal Activity (EDA)

EDA measures the electrical conductance of the skin, which is made possible by sweat glands controlled by the sympathetic nervous system. EDA was used as an objective measure of psychophysiological activity [59]. EDA was assessed utilizing a Mindware MW3000A Bio-Potential and SC Monitor. Silver-chloride cup electrodes were placed on a medial site of the inner side of the foot, over the abductor hallucis muscle, adjacent to the foot sole, and midway between the proximal phalanx of the big to and a point directly beneath the ankle as determined by best practice [60]. Data was collected with BioLab Acquisition Software and inspected visually during the experiment by either the principal investigator or a research assistant trained by the principal investigator. After the experiment, the signal was amplified 10x and processed through a 1 Hz Low Pass filter to remove artifacts caused by movement. All physiological data was then scored in EDA (Mindware Technologies, Gahanna, OH) by the principal investigator.

### Procedure

Following consent, participants were asked to complete self-report measures (described above). Participants were then equipped with the controls and VR equipment and connected to the MW3000A physiological recorder. After the experimenters ensured all systems were functioning correctly, participants were given a ten minute baseline period to familiarize themselves with the navigation controls within a non-experimental room within the VE. The participants were then informed that they would be navigating through a virtual environment as directed by narrative, and given the following set of instructions:

We are going to begin. During the experiment, we are going to present you with a virtual reality scene. Please navigate your way through the scenario as we describe it to you. Elements of the environment will be described to you in detail. Your job is to imagine yourself in the environment exactly as it is presented. Please remain focused on the scene; particularly, do not imagine anything that would make you feel more comfortable or relaxed. At certain points, you will be asked to rate how much you feel you are immersed in the environment or in other words, how much you feel you are really there. We will use the 1 to 7 point scale where 1 is “not at all” and 7 is where you feel “completely” immersed. When you are asked for your rating, try to give me the rating as truthfully and as quickly as possible. Your rating is very important. Do you have any questions before we begin? You will be notified when the experiment is over, and given further instructions. Here we go…

During Trial 1 (T1), 50% (*n* = 30) of participants received OS congruent with the VE; the other half of participants received no OS. After completion of T1, participants removed the HMD and headphones for a ten minute reset period, during which they completed additional self-report measures for T1. During the presentation of olfactory stimuli, the delivery device made slight, though audible, clicking sound as solenoids were activated (on/off) to disperse the OS. To ensure this noise did not confound our results, the delivery device was activated for all participants regardless of condition. This was achieved by routing compressed air through chambers that did or did not contain stimuli as needed. During the rest period, the air in the room was vented by fans to minimize between-trial contamination.

**Fig 1:**
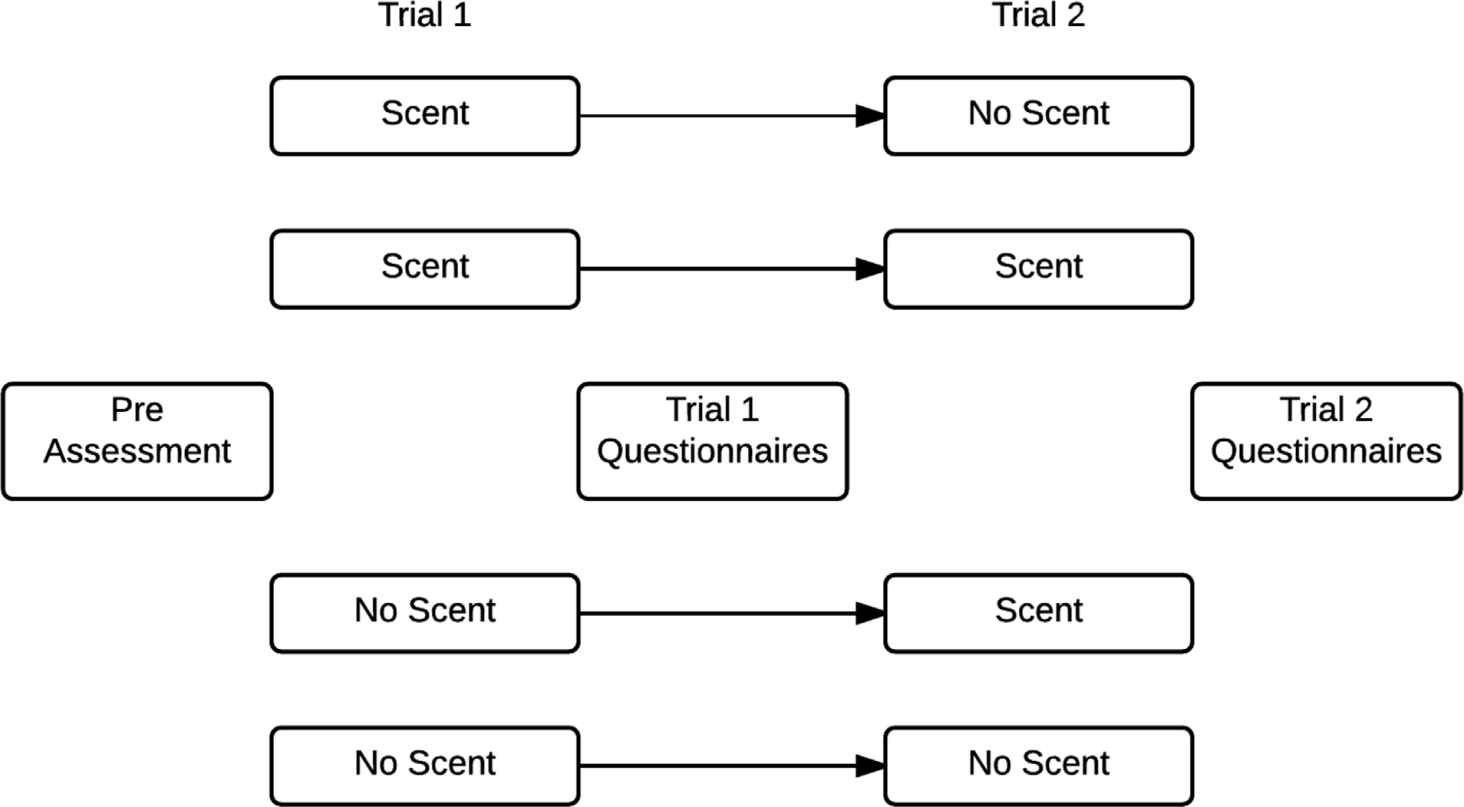
Experiment Flow.

After the reset period concluded, experiment instructions were reiterated and Trial 2 (T2) began. Trial 2 was identical to Trial 1 with the exception of experimental olfactory manipulation. During T2, 50% (*n* = 30) of the sample reversed olfactory condition, while the remaining participants remained in their T1 condition, meaning they either continued receiving scents, or they again received no scents. Upon completion of T2, participants completed self-report measures for T2. Subjects were also asked if they had ever experienced events similar to those within the VE in real life. All subjects denied autobiographical memories similar in nature to the VE, at which point their participation in the study concluded. The experiment flow can be seen in Fig 1.

### Olfactory Stimuli

**Fig 2:**
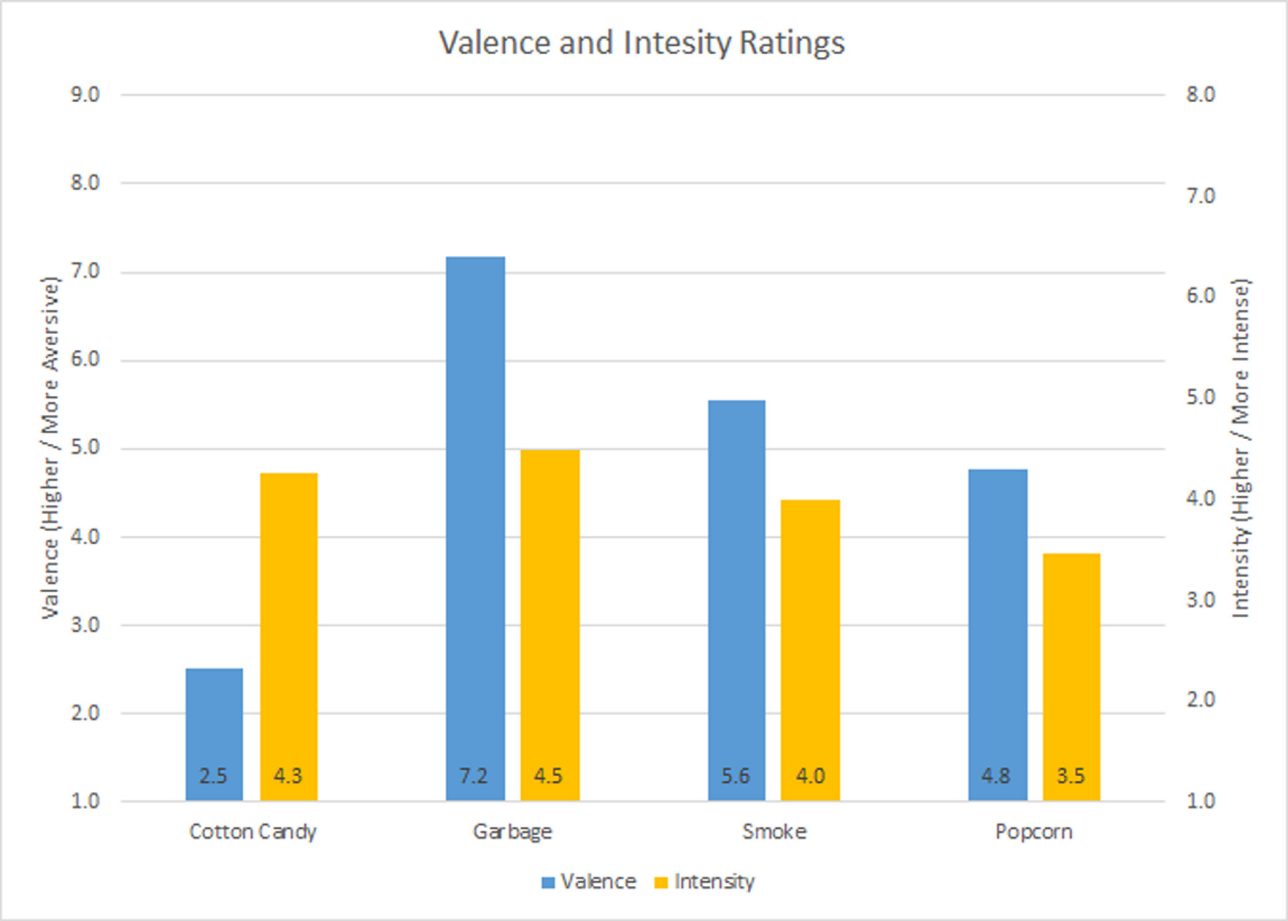
Stimuli Valence and Intensity Ratings.

The OS were ceramic pellets impregnated with scented oil (Dreamreapers Inc., Melrose Park, IL) consistent with the scent of smoke, garbage, cotton candy, and popcorn. Stimuli were delivered via air dispersion by a USB controlled Scent Palette (Virtually Better Inc., Decatur, GA), which, when activated, delivered 80 psi of compressed air through the scent chamber. Each OS dispersion had a duration of seven seconds, with each scent being presented with equal frequency throughout the VE. The chemical composition of each scent can be seen in Table 1. Safety Data Sheets (SDS, S1–S5 Files). Olfactory stimuli were selected specifically to augment the virtual environment. Valence and intensity ratings were collected from an untrained, counterbalanced, independent sample to validate valances and intensities. No attempts were made to standardize participant responses, and valance ratings were collected on a nine point Likert scale ranging from one (Very Pleasant) to nine (Offensive), while intensity ratings ranged from one (Not Detectable) to (Intolerable). Valence and intensity ratings can be seen in Fig 2.

**Table 1:** Chemical Composition of Olfactory Stimuli.

Smoke (S1 File)		Garbage	
Cade Oil (Rectified)	1–5%	Stinky Cheese (S4 File)	
Benzyl Benzoate	>80%	Benzyl Benzoate	92.56%
Popcorn (S2 File)		Butyric Acid	2.95%
Butyric Acid	0.28%	Hexanoic Acid	2%
Acetoin	2.83%	Phenylacetic Acid	1.2%
Acetyl Propionyl	4.83%	Cresyl Acetate Para	1%
		Other	0.29%
Cotton Candy (S3 File)		Garlic (S5 File)	
Benzyl Benzoate	50–65%	Trade Secret	>80%
Ethyl Vanillin	5–10%		
Ethyl Maltol	5–10%		
Coumarin	5–10%		
p-Methoxybenzaldehyde	1–5%		
B.H.T.	1–5%		

Note: The Garbage stimuli was composed of two chemical compounds as seen here.

### Virtual Reality System

The VE was modeled in 3D and controlled with the Unity3D engine (Unity Technologies, San Francisco, CA) and approximated an abandoned carnival at night. Participants were asked to imagine that they had lost their keys within the carnival, and were directed to retrieve them. The VE was presented to the subject using the Oculus Development Kit II HMD (Oculus VR, Irvine, CA) and high-fidelity stereo headphones (Audio Technica ATH-M50x; Audio Technica, Stow, Ohio). The VE was generated by a PC with an Intel^®^ i5-4670 3.4ghz CPU, 16 gigabytes of RAM, and a Nvidia^®^ GTX 780 Ti GPU. Participants navigated through the environment at their own pace and had full control over their movement utilizing a wireless Xbox 360 controller (Microsoft Inc., Redmond, WA). The participants were equipped with a virtual flashlight allowing them to explore any unlighted areas should they choose to examine the VE in greater depth. Participants were guided through the VE via location-based prerecorded narration. Congruent ambient sounds accompanied the visuals of the VE. At various locations within the VE, scripted events were presented to add realism to the VE. For example, an audio sample of an unseen object bumping into a metal garbage can was played as the participant passed a 3D garbage can. For those in the OS condition, this was augmented with the smell of garbage.

## Results

### Data screening

Of 122 adults recruited via community announcements and the University of Central Florida, 62 were not included in the final analyses. Reasons include simulator sickness and discontinuation (*n* = 18), subthreshold ability to smell (*n* = 5), technical malfunctions (*n* = 38) and noncompliance with the experimental task (*n* = 1). Chi-squares and ANOVAs were conducted to determine if those excluded from the final sample were different proportionally to those included. No significant differences were found with the exception of gender; females were more likely to report their desire to discontinue or suffer from simulator sickness than males (*p = 0*.*012*). Video game use did significantly differ by participant sex, R2 = 0.26, (F(1,52) = 15.647, p = 0.0002. However, there were no differences between groups (F(3,52) = 0.614, p = 0.608), and no significant group*sex difference was identified (F(3,52) = 0.197, p = 0.897.

Jackknife distance measures were calculated to identify multivariate outliers utilizing the critical value formula recommended in Penny [61]. Seven such outliers were identified, but demonstrated a negligible effect on *p*-values. These outliers did not possess enough influence to alter the significance of any analyses. Thus, the outliers were included in the final sample.

### Statistical analyses

All analyses were conducted on the final sample of 60 participants using JMP Pro 11.2.0 (SAS Institute Inc., Cary NC) after screening for data normalcy. All analyses defined significance utilizing a Bonferroni corrected *p*-value of < 0.01.

### Presence ratings

**Fig 3:**
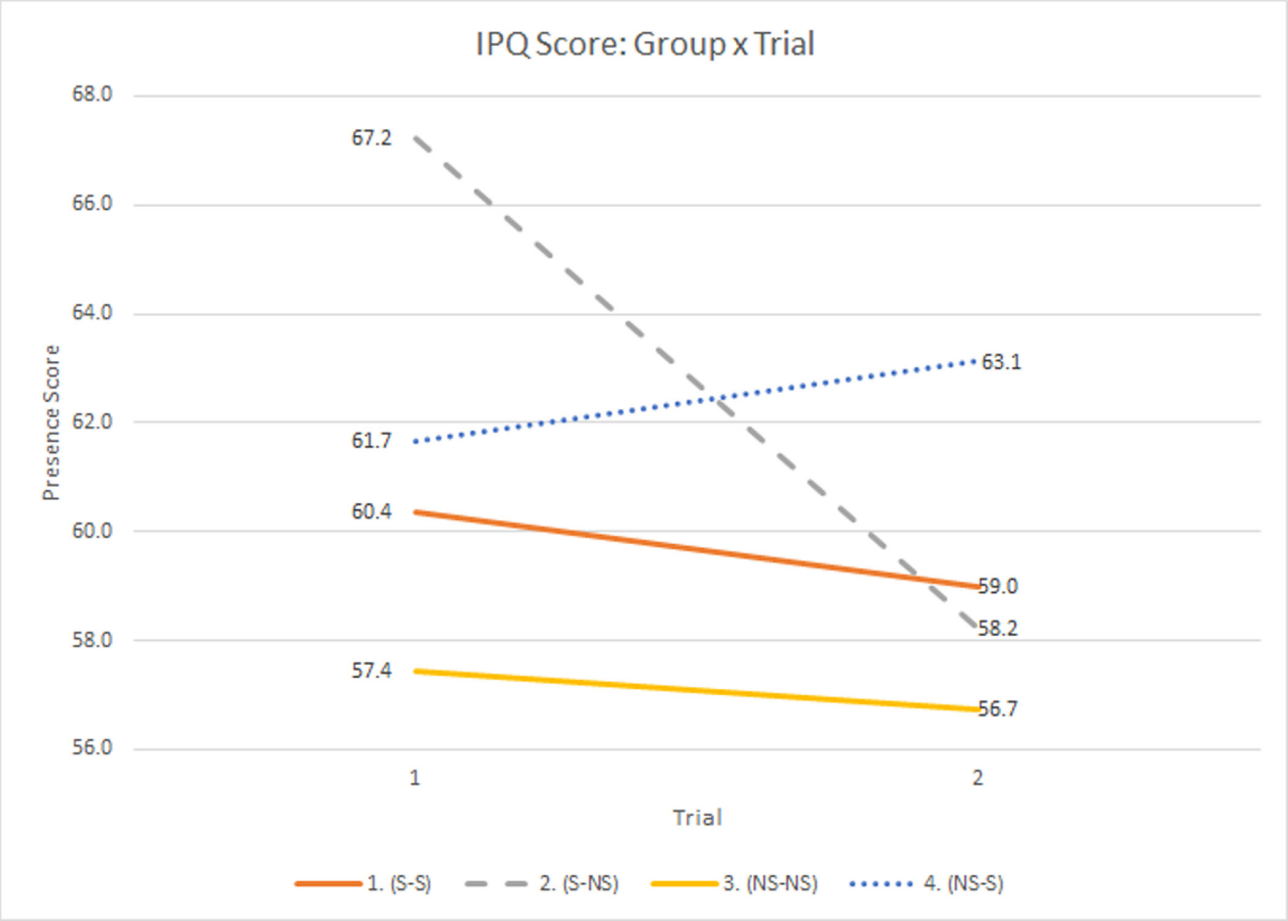
IPQ Scores by Trial.

Linear Mixed Model (LMM) analyses were utilized to assess change between trials for continuous outcome variables and within- and between-subject effects. Group membership served as a between-subjects effect, while trial and sex were assigned as within-subjects factors. IPQ scores were examined utilizing LMM predicted by sex, trial, gender, and group. There was near significant main effect for trial, *R*^2^ = 0.85, (*F*(1,52) = 6.3669, *p* = .0147), and the group*trial interaction, *R*^2^ = 0.85, (*F*(3,52) = 6.625, *p* = 0.0007). With regard to the main effect for trial, participants felt significantly more present during T1 than T2 (LSM_T1_ = 61.68 & LSM_T2_ = 59.26). With regard to the interaction (Fig 3), the Scent-No Scent (S-NS) group showed a disproportionate decrease in presence in T2 compared to other groups, while the No Scent-Scent (NS-S) group reported an increase in presence. These changes in score indicated that participants felt more presence when OS were present. The control groups maintained relative stability across trials, as the Scent-Scent (S-S) group on average declined by just over a single point (1.37, LSM_T1_ = 60.37 & LSM_T2_ = 59.00) while the No Scent-No Scent (NS-NS) group declined less than one point (0.7, LSM_T1_ = 57.44 & LSM_T2_ = 56.74). A significant effect found for sex was not significant after the Bonferroni correction, *R*^2^ = 0.85, (*F*(1,52) = 4.4623, *p* = 0.0396).

**Fig 4:**
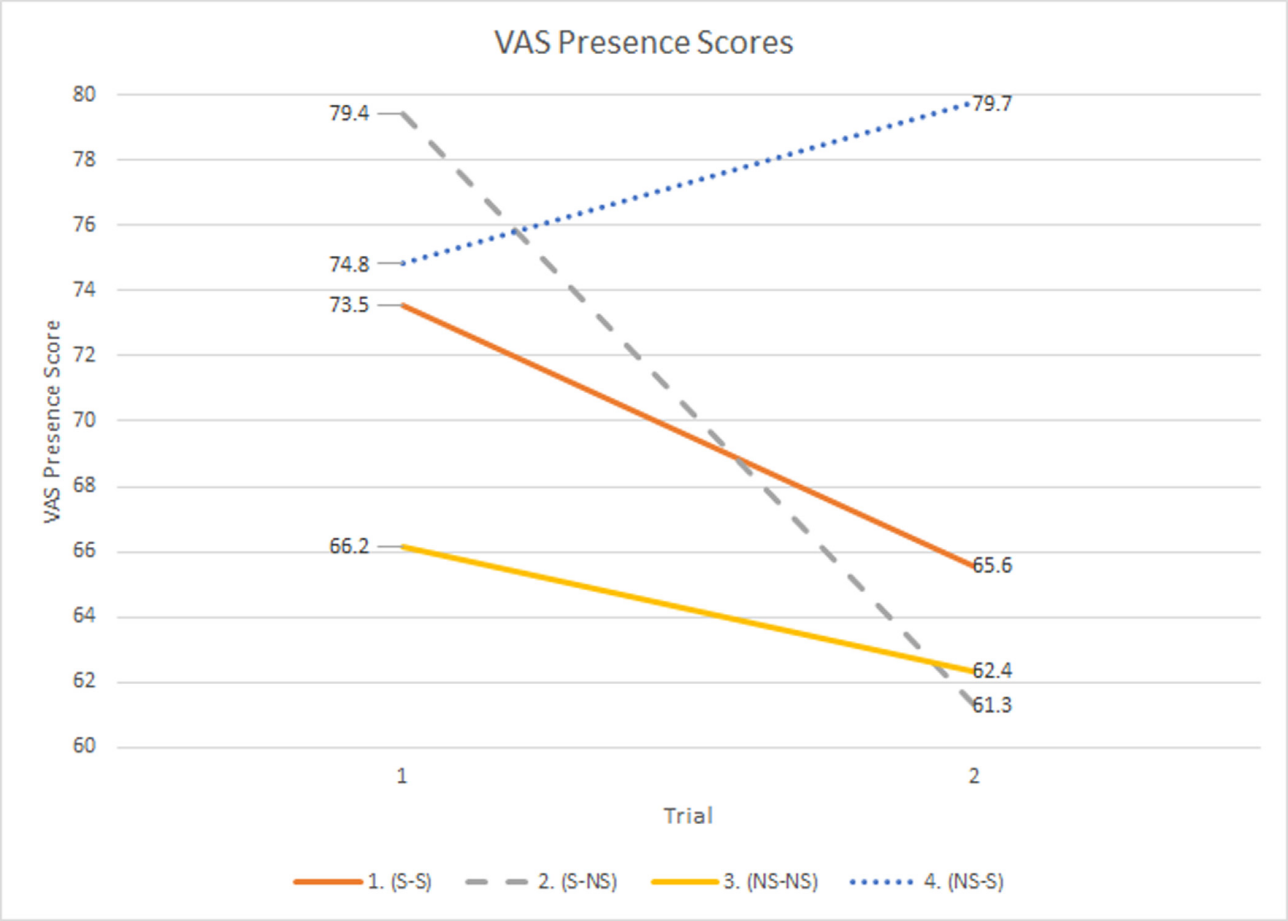
VAS Score by Trial.

Examination of the VAS showed a significant main effect for trial, *R*^2^ = 0.81, (*F*(1,52) = 7.955, *p* = 0.0068), also indicating that participants felt more present during T1 (LSM_T1_ = 73.48 & LSM_T2_ = 67.25). The group*trial interaction, *R*^2^ = 0.85, (*F*(3,52) = 5.382, *p* = 0.0027) showed a disproportionate decrease in presence between T1 and T2 (LSM_T1_ = 79.43 & LSM_T2_ = 61.32) in the S-NS group compared to other groups. As seen in Fig 4, the NS-S group saw a gain in VAS scores during T2 (LSM_T1_ = 74.81 & LSM_T2_ = 79.74) which suggests that introducing OS increased perceived presence, even after one trial experienced within the VE.

### Anxiety ratings

**Fig 5:**
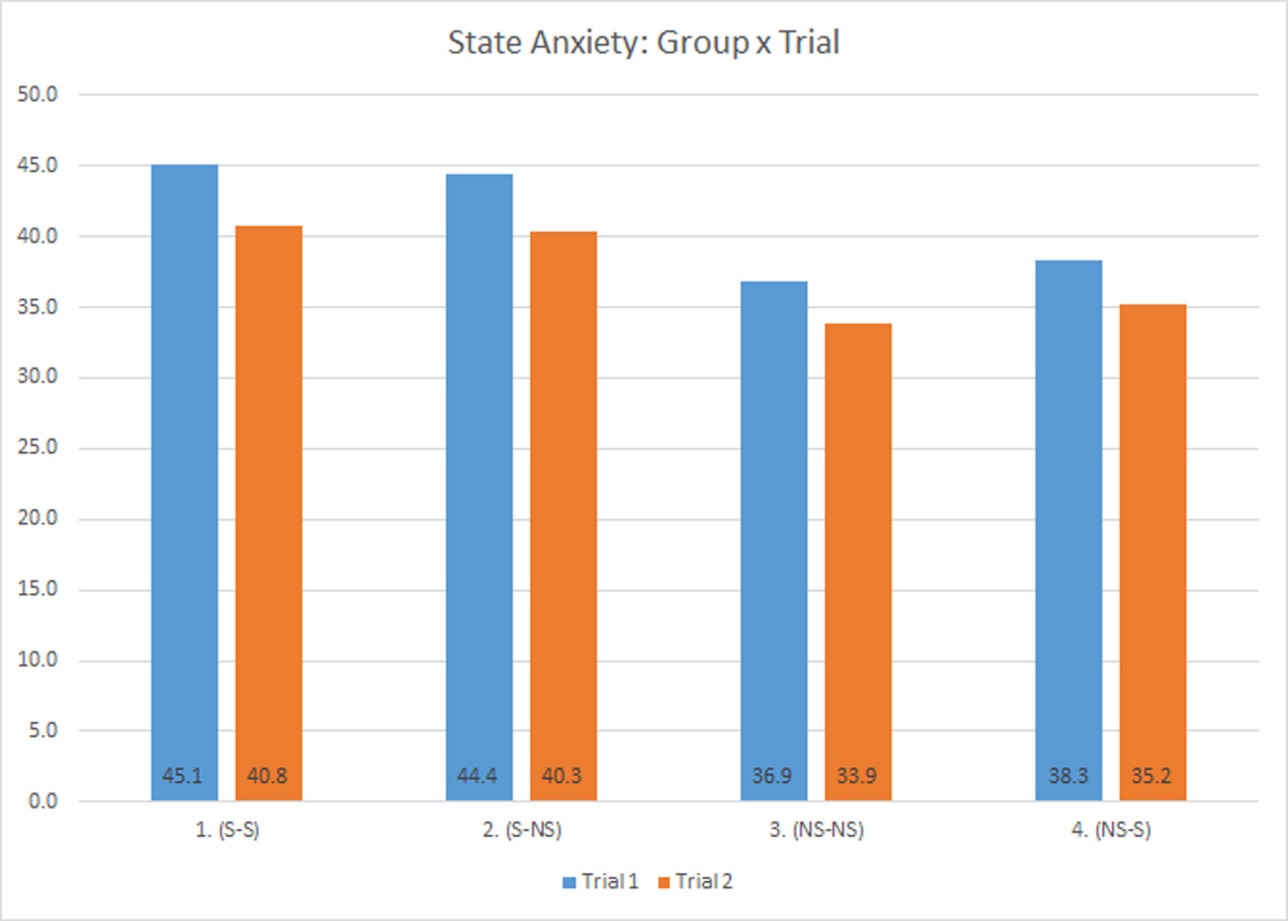
State Anxiety Scores by Trial.

State anxiety scores as reported on the STAI showed a main effect for trial, *R*^2^ = 0.76, (*F*(2,104) = 45.396, *p* < 0.0001), indicating participants felt most anxious during the first trial, with state anxiety in T1 significantly higher than at pre-exposure and T2 (LSM_PRE_ = 28.08, LSM_T1_ = 41.18, & LSM_T2_ = 37.54, Fig 5). The group*trial interaction was not statistically significant in this model (*F*(6,104) = 1.002, *p* = 0.427). These STAI scores may indicate that the VE induced mild anxiety as intended and that reported anxiety decreased between trials.

### Physiological measures

**Fig 6:**
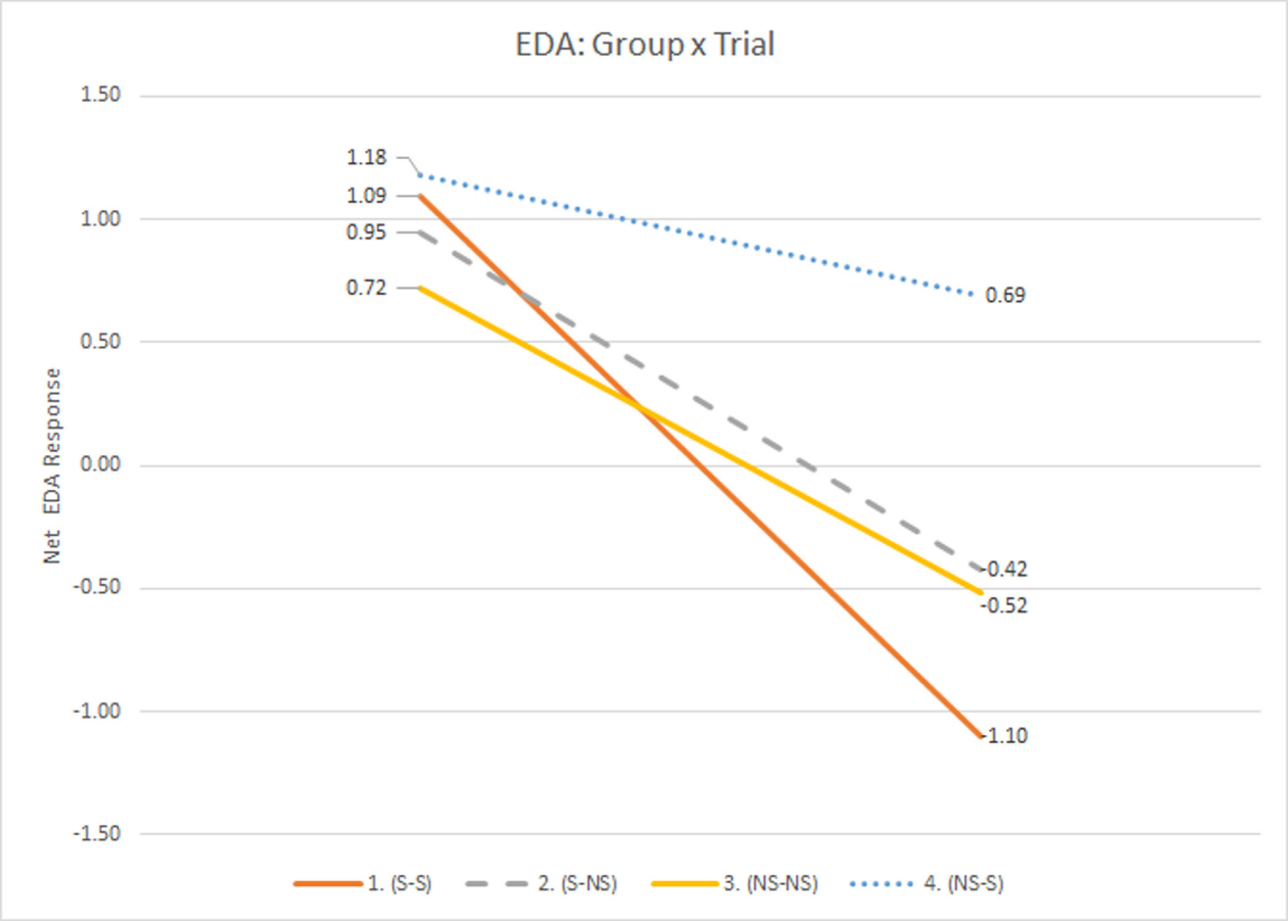
Group*Trial Interaction for EDA.

A main effect for trial (*F*(1,52) = 40.822, *p* < 0.0001) was observed when analyzing net EDA within the LMM. A group*trial interaction approached significance (*F*(3,52) = 2.600, *p* = 0.061). The main effect for trial showed participants were more aroused during Trial 1 (LSM_T1_ = 0.985_μS_ & LSM_T2_ = -0.337 _μS_). The group*trial interaction showed that the group that received olfactory stimuli in both trials had a significant reduction in arousal during the second trial (LSM_G1T1_ = 1.093_μS_ & LSM_G1T2_ = -1.099_μS_). Similarly, those who received scents during Trial 1 but not Trial 2 demonstrated a disproportionate decrease in arousal during the second trial (LSM_G2T1_ = 0.947_μS_ & LSM_G2T2_ = -0.422_μS_). These results can be seen in Fig 6.

Differences in event-related skin conductance responses (ER-SCR) and nonspecific SCRs (NS-SCR) was also examined through LMM. NS-SCRs were defined as a fluctuation greater than 0.05_μS_, while ER-SCRs were defined as a fluctuation greater than 0.05_μS_ that occurred within a three second window following a scripted event within the VE. LMM analyses of ER-SCR revealed a significant main effect for trial (*F*(1,52) = 35.883, *p* < 0.0001). Similarly, a significant main effect for trial was found for NS-SCRs (*F*(1, 52) = 75.995, *p* < 0.0001). Results indicated that participants were not as physiologically reactive to scripted events during Trial 2 (LSM_T1_ = 2.734 & LSM_T2_ = 1.84). Spontaneous reactions also decreased in Trial 2, indicating fewer spontaneous reactions during Trial 2 (LSM_T1_ = 16.960 & LSM_T2_ = 8.213).

### Condition Identification

Another variable of interest was whether or not participants would be able to correctly identify the trial condition they had just received after each VE exposure. After each trial participants were asked if they received scents or smells during the trial they had just completed. This question was evaluated with distractor items, which assessed for similar sensory stimuli (tactile feedback, temperature changes, and visuals). Agreement between the actual and perceived condition when olfactory stimuli were presented was excellent, with 98.8% of stimuli participants reporting true positives. During NS condition trials, 37% of participants reported experiencing odors, despite no odor being presented. This false positive rate is notable in its comparison to the distractor items it was administered with; 98.3% of participants reported receiving visual stimuli through the HMD during the experiment, when in fact all participants required visuals to complete the navigation task. 79.3% of participants also reported detecting changes in ambient room temperature, which was not manipulated.

## Discussion

This study investigated the effect of olfactory stimuli on participants’ sense of presence within a virtual environment similar to those used in the treatment of anxiety and trauma/stress disorders. It was hypothesized that the use of these stimuli during exposure would increase reported presence, as well as physiological arousal and self-reported anxiety. Results indicated across two separate measures (IPQ & VAS) that OS positively influenced the amount of perceived presence when administered, and that perceived presence decreased when OS were withheld. This finding supports our original hypothesis, and suggests that a) the addition of scents (presented as part of a virtual environment) may increase presence for participants during an exposure task, and b) the removal of scents, once presented, likely results in a reduction of presence. These findings are very important, as EXP is enhanced when the number of cues utilized increases [62, 63]. The inclusion of scents not only increases the number of treatment specific cues, but may also increase presence during the exposure task, a factor critical to the intervention’s effectiveness. Higher levels of presence may also increase the degree to which patients “buy in” to treatment, which is important as treatment credibility has been shown to increase treatment initiation [64].

Interestingly, the effect of OS on participant’s state anxiety was less than hypothesized, with olfaction making little difference. While the VE did increase anxiety from baseline, anxiety scores decreased between T1 & T2 regardless of condition. However, a serendipitous finding was the fact that those who received olfactory stimuli in T1 maintained higher levels of anxiety through T2, regardless of T2 condition. One possible explanation may be that the administration of scents during T1 activated another sensory modality in participants, who were thus more engaged throughout the experiment. Future research should examine if scents increase patient engagement during EXP.

Physiological measures also resulted in patterns different than hypothesized. As with state anxiety, physiological arousal was reduced in Trial 2, despite condition changes. It was noted by experimental staff during the data collection phase that many participants began to anticipate the scripted events in advance as evidenced by increasing EDA levels just prior to the event trigger being released. In these instances, most subjects experienced immediate reductions in EDA, which did not meet the definition of event-related SCR responses (which required an increase post-event). One possible silver-lining may be that events were only predictable due to experimental design; events such as those used in clinical settings (for example, explosions for combat-related PTSD patients) are often under clinician control, who can monitor the patient for anticipatory behaviors and circumvent them. It is also important to note that participants in this experiment lacked autobiographical memories associated with the VE that would be present in those with disorders such as PTSD. Thus, autobiographical memory may moderate or mediate the effectiveness of olfactory stimuli used during EXP.

One potential benefit of including OS during treatment may be increased generalization post-treatment. For example, in combat-related PTSD the scent of smoke may serve as a specific trigger. While traditional EXP may effectively reduce physiological reactivity in a patient with PTSD, the inclusion of smoke during EXP may allow broader generalization. Without scents included, everyday activities (like camping or cooking) may remain avoided at greater frequency than if congruent scents (e.g. smoke) had been included during the treatment. Conversely, it may be that scents affect the therapeutic process by facilitating memory recall of otherwise difficult-to-remember situations [65]. These results indicate that OS are not a detriment to presence and as such, the use of OS during EXP for disorders like PTSD or specific phobias should be considered. However, it appears that OS should not be discontinued once the user has experienced them due to reductions in reported presence. Additionally, OS may assist with treatment acceptability or in other words, patient “buy in” as anecdotal accounts of OS’s effectiveness has already been described in the memory literature [66].

There is burgeoning interest in the use of technology for the treatment of psychological disorders, particularly anxiety disorders and trauma/stress related disorders. The incorporation of these technologies can be expensive and require significant financial investments and clinician resources, with unknown therapeutic benefit. Specifically, does the use of these technologies add therapeutic efficacy over existing exposure-based interventions?

One approach to examining this issue of augmentation would be through the use of a randomized controlled trial (RCT), where one group receives the empirically supported treatment and the other group receives the empirically supported treatment plus the technology augmentation. RCTs are expensive and difficult to implement. An alternative strategy are basic experimental studies such as the one described here.

## Limitations

This study was not without limitations. First and foremost, this experiment was conducted with participants who were not inherently anxious about the VE being presented. For example, our participants did not possess fears specific to the VE that would have been present in a clinical population, such as warfighters who have experienced combat. While our VE did induce mild levels of anxiety in participants, the anxiety experienced by clinical populations may be qualitatively different. Thus, differences may exist between our sample and those who would typically receive exposure therapy. If participants were matched with a VE that targeted their specific feared stimuli or feared situation, greater differences may have emerged. Future research may wish to utilize a clinical population. For example, soldiers or veterans in a convoy VE may better illustrate the influence of scents (e.g., diesel fuel or exhaust) on presence.

Our groups were also not optimally balanced for gender, due to gender differences in experiencing simulator sickness [67]. Women were much more likely to voice their desire to discontinue. After careful deliberation, the decision was made to recruit males only due to disproportionate attrition.

To facilitate the mixed model design incorporated in this research, the decision to utilize the same VE for both trials may also explain nonsignificant differences seen in state anxiety and EDA responses. This limitation was inherent in this research design, and may be addressed in future research that includes both identical, and a manipulation VE, to better account for decreases in state anxiety and EDA.

Overall, this study demonstrates the potential of olfactory stimuli to augment exposure therapy’s treatment efficacy, and indicates that OS may be effective in increasing presence during anxiety provoking scenarios. The use of the term *potential* is deliberate in that this is a basic experimental study and much further research is necessary. However, this study does provide sufficient data to support the need for continued investigation. In addition to the data that adding olfaction increased presence, the score patterns for the reversal groups (S-NS & NS-S) trended in the hypothesized directions. If OS directly increases presence during individual sessions of EXP, the next steps would be attempts to replicate the study with clinical populations. Given the escalating patient care costs of mental health disorders, the utilization of scents may positively impact treatment efficacy through increased patient acceptability or greater habituation in-session. Conversely, it may be that scents affect the therapeutic process by facilitating memory recall of otherwise difficult-to-remember situations. More research in this area is required.

## Supporting Information

S1 File (PDF)

S2 File (PDF)

S3 File (PDF)

S4 File (PDF)

S5 File (PDF)
