# Supplementary material for: Olfactory Stimuli Increase Presence in Virtual Environments
Source: PLoS One. 2016 Jun 16;11(6):e0157568. doi: 10.1371/journal.pone.0157568 (PMC4910977; doi:10.1371/journal.pone.0157568)
Supplement: S1 File — (PDF) [file pone.0157568.s001.pdf]

CAMPFIRE SCENT FRAGRANCE 5276

Safety Data Sheet 8X-900-052-76

according to Federal Register / Vol. 77, No. 58 / Monday, March 26, 2012 / Rules and Regulations

Date of issue: 05/30/2015

SECTION 1: Identification of the substance/mixture and of the company/undertaking

1.1. Product identifier

Name : CAMPFIRE SCENT FRAGRANCE 5276

1.2. Relevant identified uses of the substance or mixture and uses advised against

Use of the substance/mixture : Industrial uses: Uses of substances as such or in preparations* at industrial sites

1.3. Details of the supplier of the safety data sheet

Berje
700 Blair Road
Carteret, NJ 07008 - USA
T 973-748-8980 - F 973-680-9618
berje@berjeinc.com - www.berjeinc.com

1.4. Emergency telephone number

Emergency number : 1-800-424-9300 24 Hours Emergency Chemtrec. ERR - CCN2624

SECTION 2: Hazards identification

2.1. Classification of the substance or mixture

GHS-US classification

Aquatic Acute 2	H401
Aquatic Chronic 2	H411

Full text of H-phrases: see section 16

2.2. Label elements

GHS-US labelling

Hazard pictograms (GHS-US) :

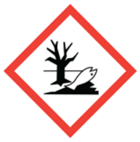

GHS09

Hazard statements (GHS-US) : H401 - Toxic to aquatic life
H411 - Toxic to aquatic life with long lasting effects

Precautionary statements (GHS-US) : P273 - Avoid release to the environment
P391 - Collect spillage
P501 - Dispose in a safe manner in accordance with local/national regulations

2.3. Other hazards

No additional information available

2.4. Unknown acute toxicity (GHS-US)

Not applicable

SECTION 3: Composition/information on ingredients

3.1. Substance

Not applicable

3.2. Mixture

Name	Product identifier	%	GHS-US classification
Benzyl benzoate	(CAS No) 120-51-4	>= 80	Acute Tox. 4 (Oral), H302<br>Aquatic Acute 2, H401<br>Aquatic Chronic 2, H411
CADE OIL RECTIFIED	(CAS No) 8013-10-3	1 - 5	Asp. Tox. 1, H304<br>Aquatic Acute 1, H400<br>Aquatic Chronic 1, H410

Full text of H-phrases: see section 16

SECTION 4: First aid measures

4.1. Description of first aid measures

First-aid measures general : Never give anything by mouth to an unconscious person. If you feel unwell, seek medical advice (show the label where possible).

First-aid measures after inhalation : Allow victim to breathe fresh air. Allow the victim to rest.

First-aid measures after skin contact : Remove affected clothing and wash all exposed skin area with mild soap and water, followed by warm water rinse.

First-aid measures after eye contact : Rinse immediately with plenty of water. Obtain medical attention if pain, blinking or redness persist.

First-aid measures after ingestion : Rinse mouth. Do NOT induce vomiting. Obtain emergency medical attention.

4.2. Most important symptoms and effects, both acute and delayed

Symptoms/injuries : Not expected to present a significant hazard under anticipated conditions of normal use.

4.3. Indication of any immediate medical attention and special treatment needed

No additional information available

SECTION 5: Firefighting measures

5.1. Extinguishing media

Suitable extinguishing media : dry chemical powder. Foam. Dry powder. Carbon dioxide. Water spray. Sand.

Unsuitable extinguishing media : Do not use a heavy water stream.

5.2. Special hazards arising from the substance or mixture

No additional information available

5.3. Advice for firefighters

Firefighting instructions : Use water spray or fog for cooling exposed containers. Exercise caution when fighting any chemical fire. Prevent fire-fighting water from entering environment.

Protection during firefighting : Do not enter fire area without proper protective equipment, including respiratory protection.

SECTION 6: Accidental release measures

6.1. Personal precautions, protective equipment and emergency procedures

6.1.1. For non-emergency personnel

Emergency procedures : Evacuate unnecessary personnel.

6.1.2. For emergency responders

Protective equipment : Equip cleanup crew with proper protection.

Emergency procedures : Ventilate area.

6.2. Environmental precautions

Prevent entry to sewers and public waters. Notify authorities if liquid enters sewers or public waters. Avoid release to the environment.

6.3. Methods and material for containment and cleaning up

Methods for cleaning up : Soak up spills with inert solids, such as clay or diatomaceous earth as soon as possible. Collect spillage. Store away from other materials.

6.4. Reference to other sections

See Heading 8. Exposure controls and personal protection.

SECTION 7: Handling and storage

7.1. Precautions for safe handling

Precautions for safe handling : Wash hands and other exposed areas with mild soap and water before eating, drinking or smoking and when leaving work. Provide good ventilation in process area to prevent formation of vapour.

7.2. Conditions for safe storage, including any incompatibilities

Storage conditions : Keep only in the original container in a cool, well ventilated place away from : Keep container closed when not in use.

Incompatible products : Strong bases. Strong acids.

Incompatible materials : Sources of ignition. Direct sunlight.

7.3. Specific end use(s)

No additional information available

SECTION 8: Exposure controls/personal protection

8.1. Control parameters

CAMPFIRE SCENT FRAGRANCE 5276	
ACGIH	Not applicable
OSHA	Not applicable

CADE OIL RECTIFIED (8013-10-3)	
ACGIH	Not applicable
OSHA	Not applicable

Benzyl benzoate (120-51-4)	
ACGIH	Not applicable
OSHA	Not applicable

8.2. Exposure controls

Personal protective equipment : Avoid all unnecessary exposure.

Hand protection : Wear protective gloves/protective clothing/eye protection protective gloves.

Eye protection : Chemical goggles or safety glasses.

Respiratory protection : Wear appropriate mask.

Other information : Do not eat, drink or smoke during use.

SECTION 9: Physical and chemical properties

9.1. Information on basic physical and chemical properties

Physical state : Liquid

Colour : Colourless

Odour : characteristic

Odour threshold : No data available

pH : No data available

Melting point : No data available

Freezing point : No data available

Boiling point : No data available

Flash point : > 200 °F

Relative evaporation rate (butylacetate=1) : No data available

Flammability (solid, gas) : No data available

Explosive limits : No data available

Explosive properties : No data available

Oxidising properties : No data available

Vapour pressure : No data available

Relative density : 1.09

Relative vapour density at 20 °C : No data available

Solubility : Water: Solubility in water of component(s) of the mixture :
• Benzyl benzoate: 153 mg/l (20 °C)

Log Pow : No data available

Log Kow : No data available

Auto-ignition temperature : No data available

Decomposition temperature	:	No data available
Viscosity	:	No data available
Viscosity, kinematic	:	No data available
Viscosity, dynamic	:	No data available

9.2. Other information

No additional information available

SECTION 10: Stability and reactivity

10.1. Reactivity

No additional information available

10.2. Chemical stability

Not established.

10.3. Possibility of hazardous reactions

Not established.

10.4. Conditions to avoid

Direct sunlight. Extremely high or low temperatures.

10.5. Incompatible materials

Strong acids. Strong bases.

10.6. Hazardous decomposition products

fume. Carbon monoxide. Carbon dioxide.

SECTION 11: Toxicological information

11.1. Information on toxicological effects

Acute toxicity : Not classified

**Benzyl benzoate (120-51-4)**	
LD50 oral rat	1870 mg/kg (Rat; OECD 401: Acute Oral Toxicity; Experimental value; >2000 mg/kg bodyweight; Rat)
LD50 dermal rat	4400 mg/kg (Rat)
LD50 dermal rabbit	4000 mg/kg (Rabbit; Experimental value; Modification of Draize 1959 method; >2; Rabbit)
ATE US (oral)	1870.000 mg/kg bodyweight
ATE US (dermal)	4000.000 mg/kg bodyweight

Skin corrosion/irritation	:	Not classified
Serious eye damage/irritation	:	Not classified
Respiratory or skin sensitisation	:	Not classified
Germ cell mutagenicity	:	Not classified<br>Based on available data, the classification criteria are not met
Carcinogenicity	:	Not classified
Reproductive toxicity	:	Not classified<br>Based on available data, the classification criteria are not met
Specific target organ toxicity (single exposure)	:	Not classified
Specific target organ toxicity (repeated exposure)	:	Not classified
Aspiration hazard	:	Not classified
Potential adverse human health effects and symptoms	:	Based on available data, the classification criteria are not met.

SECTION 12: Ecological information

12.1. Toxicity

Ecology - water : Toxic to aquatic life with long lasting effects.

Benzyl benzoate (120-51-4)	
LC50 fish 1	4.6 mg/l (96 h; Pisces; GLP)
EC50 Daphnia 1	426 mg/l (24 h; Daphnia magna; Locomotor effect)
LC50 fish 2	2.32 mg/l (96 h; Brachydanio rerio)
EC50 Daphnia 2	309 mg/l (48 h; Daphnia magna; Locomotor effect)
Threshold limit algae 1	647 mg/l (72 h; Pseudokirchneriella subcapitata; Biomass)
Threshold limit algae 2	247 mg/l (72 h; Pseudokirchneriella subcapitata; Growth rate)

12.2. Persistence and degradability

CAMPFIRE SCENT FRAGRANCE 5276	
Persistence and degradability	May cause long-term adverse effects in the environment.

CADE OIL RECTIFIED (8013-10-3)	
Persistence and degradability	May cause long-term adverse effects in the environment.

Benzyl benzoate (120-51-4)	
Persistence and degradability	Readily biodegradable in water. Low potential for mobility in soil.

12.3. Bioaccumulative potential

CAMPFIRE SCENT FRAGRANCE 5276	
Bioaccumulative potential	Not established.

CADE OIL RECTIFIED (8013-10-3)	
Bioaccumulative potential	Not established.

Benzyl benzoate (120-51-4)	
BCF fish 1	2286 (Pisces)
Log Pow	3.88 - 4
Bioaccumulative potential	Low potential for bioaccumulation (Log Kow < 4).

12.4. Mobility in soil

Benzyl benzoate (120-51-4)	
Surface tension	0.027 N/m (210 °C)

12.5. Other adverse effects

Effect on the global warming : No known ecological damage caused by this product.

Other information : Avoid release to the environment.

SECTION 13: Disposal considerations

13.1. Waste treatment methods

Waste disposal recommendations : Dispose in a safe manner in accordance with local/national regulations. Dispose in a safe manner in accordance with local/national regulations.

Ecology - waste materials : Avoid release to the environment.

SECTION 14: Transport information

Department of Transportation (DOT) (Non Bulk only)

In accordance with DOT

Not regulated for transport

Additional information

Other information : No supplementary information available.

ADR

Transport document description : UN 3082 ENVIRONMENTALLY HAZARDOUS SUBSTANCE, LIQUID, N.O.S. (BENZYL BENZOATE), 9, III, (E)

Packing group (ADR) : III

Class (ADR) : 9 - Miscellaneous dangerous substances and articles

Hazard identification number (Kemler No.) : 90

Classification code (ADR) : M6

Danger labels (ADR)	:	9 - Miscellaneous dangerous substances and articles

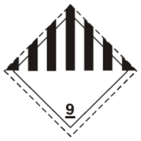

Orange plates :

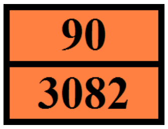

Tunnel restriction code (ADR)	:	E
Limited quantities (ADR)	:	5l
Excepted quantities (ADR)	:	E1

Transport by sea

UN-No. (IMDG)	:	3082
Proper Shipping Name (IMDG)	:	ENVIRONMENTALLY HAZARDOUS SUBSTANCE, LIQUID, N.O.S.
Class (IMDG)	:	9 - Miscellaneous dangerous substances and articles
Packing group (IMDG)	:	III - substances presenting low danger

Air transport

UN-No.(IATA)	:	3082
Proper Shipping Name (IATA)	:	Environmentally hazardous substance, liquid, n.o.s.
Class (IATA)	:	9 - Miscellaneous Dangerous Goods
Packing group (IATA)	:	III - Minor Danger

SECTION 15: Regulatory information

15.1. US Federal regulations

**CADE OIL RECTIFIED (8013-10-3)**
Listed on the United States TSCA (Toxic Substances Control Act) inventory

**Benzyl benzoate (120-51-4)**
Listed on the United States TSCA (Toxic Substances Control Act) inventory

15.2. International regulations

CANADA
No additional information available

EU-Regulations
No additional information available

Classification according to Regulation (EC) No. 1272/2008 [CLP]
Aquatic Chronic 2 H411
Full text of H-phrases: see section 16

Classification according to Directive 67/548/EEC [DSD] or 1999/45/EC [DPD]
No additional information available

National regulations
No additional information available

15.3. US State regulations

SECTION 16: Other information

Data sources	:	REGULATION (EC) No 1272/2008 OF THE EUROPEAN PARLIAMENT AND OF THE COUNCIL of 16 December 2008 on classification, labelling and packaging of substances and mixtures, amending and repealing Directives 67/548/EEC and 1999/45/EC, and amending Regulation (EC) No 1907/2006.

Other information : None.

Full text of H-phrases:

Acute Tox. 4 (Oral)	Acute toxicity (oral), Category 4
Aquatic Acute 1	Hazardous to the aquatic environment — Acute Hazard, Category 1
Aquatic Acute 2	Hazardous to the aquatic environment — Acute Hazard, Category 2
Aquatic Chronic 1	Hazardous to the aquatic environment — Chronic Hazard, Category 1
Aquatic Chronic 2	Hazardous to the aquatic environment — Chronic Hazard, Category 2
Asp. Tox. 1	Aspiration hazard, Category 1
H302	Harmful if swallowed
H304	May be fatal if swallowed and enters airways
H400	Very toxic to aquatic life
H401	Toxic to aquatic life
H410	Very toxic to aquatic life with long lasting effects
H411	Toxic to aquatic life with long lasting effects

HMIS III Rating

Health : 1 Slight Hazard - Irritation or minor reversible injury possible

Flammability : 1 Slight Hazard - Materials that must be preheated before ignition will occur. Includes liquids, solids and semi solids having a flash point above 200 F. (Class IIIB)

Physical : 0 Minimal Hazard - Materials that are normally stable, even under fire conditions, and will NOT react with water, polymerize, decompose, condense, or self-react. Non-Explosives.

Personal Protection : B

B - Safety glasses, Gloves

SDS US (Berje)

The information contained in this Safety Data Sheet (SDS) was obtained from current and reliable sources. This data is supplied with out warranty expressed or implied, regarding its correctness or accuracy. It is the user's responsibility to determine safe conditions for use of this product and to assume liability for loss, injury, damage or expense resulting from improper use of this product
