# Supplementary material for: Olfactory Stimuli Increase Presence in Virtual Environments
Source: PLoS One. 2016 Jun 16;11(6):e0157568. doi: 10.1371/journal.pone.0157568 (PMC4910977; doi:10.1371/journal.pone.0157568)
Supplement: S2 File — (PDF) [file pone.0157568.s002.pdf]

# MATERIAL SAFETY DATA SHEET

NAME OF PRODUCT

**FILE NO.:** ORC1301387
**MSDS DATE:** 10/9/2013

**SECTION 1: PRODUCT AND COMPANY IDENTIFICATION**

**PRODUCT NAME:** Buttered Popcorn WS Fragrance F# ORC1301387
**SYNONYMS:** N/A
**PRODUCT CODES:** ORC1301387

**MANUFACTURER:** Orchidia Fragrances, a division of Flavorchem Corporation
**DIVISION:**
**ADDRESS:** 1525 Brook Drive, Downers Grove, IL 60515

**EMERGENCY PHONE:** Domestic North America 800-424-9300, International 703-527-3887
**CHEMTREC PHONE:** Domestic North America 800-424-9300, International 703-527-3887
**OTHER CALLS:**
**FAX PHONE:**

**CHEMICAL NAME:** N/A
**CHEMICAL FAMILY:** N/A
**CHEMICAL FORMULA:** N/A

**PRODUCT USE:**
**PREPARED BY:** Orchidia Fragrances, a division of Flavorchem Corporation

**SECTION 1 NOTES:**

**SECTION 2: COMPOSITION/INFORMATION ON INGREDIENTS**

| HAZARDOUS INGREDIENTS: | CAS NO. | % WT | % VOL | SARA 313 REPORTABLE |
|---|---|---|---|---|
| Butyric Acid | 107-92-6 | 0.28 | | |
| Acetoin | 513-86-0 | 2.82 | | |
| Acetyl Propionyl | 600-14-6 | 4.83 | | |

| | ppm | mg/m3 |
|---|---|---|
| OSHA PEL | | |
| Butyric Acid | N/A | |
| Acetoin | N/A | |
| Acetyl Propionyl | N/A | |
| ACGIH TLV | | |
| Butyric Acid | N/A | |
| Acetoin | N/A | |
| Acetyl Propionyl | N/A | |

**SECTION 2 NOTES:**

**SECTION 3: HAZARDS IDENTIFICATION**

**EMERGENCY OVERVIEW:**

**ROUTES OF ENTRY:** Eyes, skin, ingestion, inhalation

**POTENTIAL HEALTH EFFECTS**

**EYES:** May cause eye irritation by direct contact or from fumes.

**SKIN:** Prolonged contact may lead to irritation and dermatitis.

**INGESTION:** Harmful if swallowed in concentrated form.

**INHALATION:** May cause respiratory tract irritation.

**ACUTE HEALTH HAZARDS:** Not determined.

**CHRONIC HEALTH HAZARDS:** Not determined.

**MEDICAL CONDITIONS GENERALLY AGGRAVATED BY EXPOSURE:** Not determined.

**CARCINOGENICITY**
**OSHA: ACGIH: NTP: IARC:**
**OTHER:**
**Not determined.**

**SECTION 3 NOTES:**

## SECTION 4: FIRST AID MEASURES

**EYES: Flush eyes with water for at least 15 minutes. If irritation persists, contact a doctor.**

**SKIN: Wash skin with soap and plenty of water. Remove contaminated clothing and wash before wearing again.**

**INGESTION: If swallowed, drink milk or water and contact a doctor immediately.**

**INHALATION: Remove victim to fresh air.**

**NOTES TO PHYSICIANS OR FIRST AID PROVIDERS:**

**SECTION 4 NOTES:**

## SECTION 5: FIRE-FIGHTING MEASURES

**FLAMMABLE LIMITS IN AIR, UPPER: Not known**
**(% BY VOLUME) LOWER: Not known**

**FLASH POINT:**
**F: 174 deg F**
**C: 78.89 deg C**
**METHOD USED: Closed cup**

**AUTOIGNITION TEMPERATURE:**
**F: Not determined**
**C: Not determined**

**NFPA HAZARD CLASSIFICATION**
**HEALTH: 1 FLAMMABILITY: 2 REACTIVITY: 0**
**OTHER:**

**HMIS HAZARD CLASSIFICATION**
**HEALTH: FLAMMABILITY: REACTIVITY:**
**PROTECTION:**

**EXTINGUISHING MEDIA: Carbon dioxide, dry chemical or universal-type foam applied according to manufacturer's techniques. Water spray.**

**SPECIAL FIRE FIGHTING PROCEDURES: Wear self-contained breathing apparatus and complete personal protective equipment.**

**UNUSUAL FIRE AND EXPLOSION HAZARDS: Closed containers may build up pressure at elevated temperatures. If possible, containers should be coolded with a water spray.**

**HAZARDOUS DECOMPOSITION PRODUCTS: Burning can produce carbon dioxide and/or carbon monoxide.**

**SECTION 5 NOTES:**

## SECTION 6: ACCIDENTAL RELEASE MEASURES

**ACCIDENTAL RELEASE MEASURES:**
**Small Spill: Cover with a non-combustible material and remove to an approved disposal container. Wash area with soap and water.**
**Large Spill: Avoid sources of ignition. Contain spilled material. Cover with a non-combustible material and remove to an approved disposal container. Avoid inhalation of vapors. If in a confined area, NIOSH approved respiratory protection may be**

**required. Wash area with soap and water. Dry products should be swept up and removed to an approved disposal container. Wash area with soap and water.**

**SECTION 6 NOTES:**

## SECTION 7: HANDLING AND STORAGE

**HANDLING AND STORAGE: Store in tightly sealed containers in a cool dry place that is well ventilated.**

**OTHER PRECAUTIONS:**

**SECTION 7 NOTES:**

## SECTION 8: EXPOSURE CONTROLS/PERSONAL PROTECTION

**ENGINEERING CONTROLS: Not determined.**

**VENTILATION : Provide adequate ventilation using a mechanical exhaust fan.**

**RESPIRATORY PROTECTION: In well ventilated areas, respiratory protection is not normally required. In poorly ventilated areas, use NIOSH respirator.**

**EYE PROTECTION: Chemical safety goggles recommended.**

**SKIN PROTECTION: Chemical resistant gloves recommended.**

**OTHER PROTECTIVE CLOTHING OR EQUIPMENT: Eye wash station and safety shower should be easily accessible.**

**WORK HYGIENIC PRACTICES: Not determined.**

**EXPOSURE GUIDELINES: Not determined.**

**SECTION 8 NOTES:**

## SECTION 9: PHYSICAL AND CHEMICAL PROPERTIES

**APPEARANCE: A clear yellow liquid**

**ODOR: Buttered popcorn**

**PHYSICAL STATE: Liquid**

**pH AS SUPPLIED: Not available**
**pH (Other): Not available**
**BOILING POINT: Not available**
**F:**
**C:**

**MELTING POINT: Not available**
**F:**
**C:**

**FREEZING POINT: Not available**
**F:**
**C:**

**VAPOR PRESSURE (mmHg): Not available**
**@**
**F:**
**C:**

**VAPOR DENSITY (AIR = 1): Heavier**
**@**
**F:**
**C:**

**SPECIFIC GRAVITY (H2O = 1): 1.09 (+/-0.05)**
@
**F: 77 deg.**
**C: 25 deg.**

**EVAPORATION RATE: Not available**

**BASIS (=1): Not available**

**SOLUBILITY IN WATER: Soluble**

**PERCENT SOLIDS BY WEIGHT: Not available**

**PERCENT VOLATILE: Not available**

**BY WT/ BY VOL @**
**F:**
**C:**
**VOLATILE ORGANIC COMPOUNDS (VOC): Not available**

**WITH WATER: LBS/GAL**
**WITHOUT WATER: LBS/GAL**

**MOLECULAR WEIGHT: Not available**

**VISCOSITY: Not available**
@
**F:**
**C:**

**SECTION 9 NOTES:**

## SECTION 10: STABILITY AND REACTIVITY

**STABILITY: Stable.**

**CONDITIONS TO AVOID (STABILITY): Avoid heat, sparks and open flames.**

**INCOMPATIBILITY (MATERIAL TO AVOID): Avoid contact with strong alkalies and oxidizing agents.**

**HAZARDOUS DECOMPOSITION OR BY-PRODUCTS: Burning can produce carbon dioxide and/or carbon monoxide.**

**HAZARDOUS POLYMERIZATION: Will not occur.**

**CONDITIONS TO AVOID (POLYMERIZATION):**

**SECTION 10 NOTES:**

## SECTION 11: TOXICOLOGICAL INFORMATION

**TOXICOLOGICAL INFORMATION: Not determined**

**SECTION 11 NOTES:**

## SECTION 12: ECOLOGICAL INFORMATION

**ECOLOGICAL INFORMATION: Not determined**

**SECTION 12 NOTES:**

## SECTION 13: DISPOSAL CONSIDERATIONS

**WASTE DISPOSAL METHOD: Incinerate, bury in approved manner or dispose of using any procedure which is in conformance with pertinent federal, state or local regulations.**

**RCRA HAZARD CLASS:**

**SECTION 13 NOTES:**

## SECTION 14: TRANSPORT INFORMATION

**U.S. DEPARTMENT OF TRANSPORTATION Not regulated**
**PROPER SHIPPING NAME:**
**HAZARD CLASS:**
**ID NUMBER:**
**PACKING GROUP:**
**LABEL STATEMENT:**

**WATER TRANSPORTATION Not regulated**
**PROPER SHIPPING NAME:**
**HAZARD CLASS:**
**ID NUMBER:**
**PACKING GROUP:**
**LABEL STATEMENTS:**

**AIR TRANSPORTATION Not regulated**
**PROPER SHIPPING NAME:**
**HAZARD CLASS:**
**ID NUMBER:**
**PACKING GROUP:**
**LABEL STATEMENTS:**

**OTHER AGENCIES:**

**SECTION 14 NOTES:**

## SECTION 15: REGULATORY INFORMATION

**U.S. FEDERAL REGULATIONS**
**TSCA (TOXIC SUBSTANCE CONTROL ACT): Yes**

**CERCLA (COMPREHENSIVE RESPONSE COMPENSATION, AND LIABILITY ACT): Not determined**

**SARA TITLE III (SUPERFUND AMENDMENTS AND REAUTHORIZATION ACT): Not determined**

**311/312 HAZARD CATEGORIES: Not determined**

**313 REPORTABLE INGREDIENTS: Not determined**

**STATE REGULATIONS: Not determined**

**INTERNATIONAL REGULATIONS: Not determined**

**SECTION 15 NOTES:**

## SECTION 16: OTHER INFORMATION

**OTHER INFORMATION:**

**PREPARATION INFORMATION:**

**DISCLAIMER: The information contained herein is furnished without warranty of any kind. Employers should use this information only as a supplement to other information gathered by them and must make independent determinations of suitability and completeness of information from all sources to assure proper use of these materials and the safety and health of employees.**
