# Supplementary material for: Olfactory Stimuli Increase Presence in Virtual Environments
Source: PLoS One. 2016 Jun 16;11(6):e0157568. doi: 10.1371/journal.pone.0157568 (PMC4910977; doi:10.1371/journal.pone.0157568)
Supplement: S4 File — (PDF) [file pone.0157568.s004.pdf]

Arylessence
the essence of your brand®

SAFETY DATA SHEET

1. Identification

**Product identifier**	**AA052201 N & A Stinky Cheese Flavor**
**Other means of identification**	
**Product Code**	AA052201
**Recommended use**	Not available.
**Recommended restrictions**	None known.

Manufacturer/Importer/Supplier/Distributor information

Manufacturer

**Company name**	Arylessence, Inc.	
**Address**	1091 Lake Drive<br>Marietta, GA 30066-1073<br>United States	
**Telephone**	General Assistance	770-924-3775
**E-mail**	regulatory2@arylessence.com	
**Emergency phone number**	for shipping/transportation	spill emergencies only
	InfoTrac	800-535-5053 in the US only
	InfoTrac (International)	352-323-3500

2. Hazard(s) identification

Classification According to 29 CFR 1910.1200

**Physical hazards**	Not classified.	
**Health hazards**	Acute toxicity, oral	Category 4
	Acute toxicity, dermal	Category 4
	Acute toxicity, inhalation	Category 4
	Skin corrosion/irritation	Category 2
	Serious eye damage/eye irritation	Category 1
**Environmental hazards**	Hazardous to the aquatic environment, acute hazard	Category 1
	Hazardous to the aquatic environment, long-term hazard	Category 2
**OSHA defined hazards**	Not classified.	

Label elements

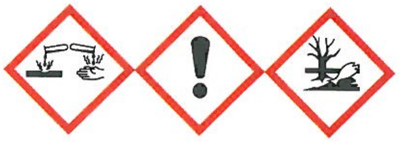

Signal word Danger

Hazard statement Harmful if swallowed. Harmful in contact with skin. Causes skin irritation. Causes serious eye damage. Harmful if inhaled. Very toxic to aquatic life. Toxic to aquatic life with long lasting effects.

Precautionary statement

Prevention Avoid breathing vapors. Wash thoroughly after handling. Do not eat, drink or smoke when using this product. Use only outdoors or in a well-ventilated area. Avoid release to the environment. Wear protective gloves/protective clothing. Wear eye/face protection.

Response If swallowed: Call a poison center/doctor if you feel unwell. If on skin: Wash with plenty of water. If inhaled: Remove person to fresh air and keep comfortable for breathing. If in eyes: Rinse cautiously with water for several minutes. Remove contact lenses, if present and easy to do. Continue rinsing. Immediately call a poison center/doctor. Rinse mouth. If skin irritation occurs: Get medical advice/attention. Take off contaminated clothing and wash before reuse. Collect spillage.

Storage Store away from incompatible materials.

**Disposal**	Dispose of contents/container in accordance with local/regional/national/international regulations.
**Hazard(s) not otherwise classified (HNOC)**	Avoid eye contact or breathing dust, powder, mist or vapor from this product as irritation of the eyes or respiratory tract may result. This product contains ingredients for which the health effects have not been fully evaluated.
**Supplemental information**	92.56% of the mixture consists of component(s) of unknown acute inhalation toxicity.

3. Composition/information on ingredients

Mixtures

Chemical name	Common name and synonyms	CAS number	%
BENZYL BENZOATE		120-51-4	92.56
BUTYRIC ACID		107-92-6	2.95
Hexanoic acid		142-62-1	2
PHENYLACETIC ACID		103-82-2	1.2
Cresyl Acetate Para- (*)		140-39-6	1
Other components below reportable levels			0.29

*Designates that a specific chemical identity and/or percentage of composition has been withheld as a trade secret.

4. First-aid measures

**Inhalation**	Remove victim to fresh air and keep at rest in a position comfortable for breathing. Oxygen or artificial respiration if needed. If experiencing respiratory symptoms: Call a POISON CENTER or doctor/physician.
**Skin contact**	Remove contaminated clothing. Rinse skin with water/shower. Get medical advice/attention if you feel unwell. If skin irritation occurs: Get medical advice/attention. Wash contaminated clothing before reuse.
**Eye contact**	Immediately flush eyes with plenty of water for at least 15 minutes. Remove contact lenses, if present and easy to do. Continue rinsing. Get medical attention immediately.
**Ingestion**	Rinse mouth. If vomiting occurs, keep head low so that stomach content doesn't get into the lungs. Get medical advice/attention if you feel unwell.
**Most important symptoms/effects, acute and delayed**	Severe eye irritation. Symptoms may include stinging, tearing, redness, swelling, and blurred vision. Permanent eye damage including blindness could result. Skin irritation. May cause redness and pain.
**Indication of immediate medical attention and special treatment needed**	Provide general supportive measures and treat symptomatically. In case of shortness of breath, give oxygen. Keep victim warm. Keep victim under observation. Symptoms may be delayed.
**General information**	Ensure that medical personnel are aware of the material(s) involved, and take precautions to protect themselves. Show this safety data sheet to the doctor in attendance.

5. Fire-fighting measures

**Suitable extinguishing media**	Water fog. Foam. Dry chemical powder. Carbon dioxide (CO_2).
**Unsuitable extinguishing media**	Do not use water jet as an extinguisher, as this will spread the fire.
**Specific hazards arising from the chemical**	During fire, gases hazardous to health may be formed.
**Special protective equipment and precautions for firefighters**	Self-contained breathing apparatus and full protective clothing must be worn in case of fire.
**Fire fighting equipment/instructions**	Move containers from fire area if you can do so without risk.
**Specific methods**	Use standard firefighting procedures and consider the hazards of other involved materials.
**General fire hazards**	No unusual fire or explosion hazards noted.

6. Accidental release measures

**Personal precautions, protective equipment and emergency procedures**	Keep unnecessary personnel away. Keep people away from and upwind of spill/leak. Wear appropriate protective equipment and clothing during clean-up. Avoid inhalation of vapor, fumes, dust and/or mist from the spilled material. Do not touch damaged containers or spilled material unless wearing appropriate protective clothing. Ensure adequate ventilation. Local authorities should be advised if significant spillages cannot be contained. For personal protection, see section 8 of the SDS.

**Methods and materials for containment and cleaning up**	Large Spills: Stop the flow of material, if this is without risk. Dike the spilled material, where this is possible. Cover with plastic sheet to prevent spreading. Absorb in vermiculite, dry sand or earth and place into containers. Prevent product from entering drains. Following product recovery, flush area with water.<br><br>Small Spills: Wipe up with absorbent material (e.g. cloth, fleece). Clean surface thoroughly to remove residual contamination.<br><br>Never return spills to original containers for re-use. For waste disposal, see section 13 of the SDS.
**Environmental precautions**	Avoid release to the environment. Prevent further leakage or spillage if safe to do so. Avoid discharge into drains, water courses or onto the ground. Inform appropriate managerial or supervisory personnel of all environmental releases.

7. Handling and storage

**Precautions for safe handling**	Do not get this material in contact with eyes. Avoid inhalation of vapors and spray mists. Avoid contact with eyes, skin, and clothing. Do not taste or swallow. When using, do not eat, drink or smoke. Use only outdoors or in a well-ventilated area. Wear appropriate personal protective equipment. Wash hands thoroughly after handling. Avoid release to the environment. Wash contaminated clothing before reuse. Observe good industrial hygiene practices.
**Conditions for safe storage, including any incompatibilities**	Store locked up. Store in original tightly closed container. Store in a well-ventilated place. Store away from incompatible materials (see Section 10 of the SDS).

8. Exposure controls/personal protection

**Occupational exposure limits**	No exposure limits noted for ingredient(s).
**Biological limit values**	No biological exposure limits noted for the ingredient(s).
**Appropriate engineering controls**	Good general ventilation (typically 10 air changes per hour) should be used. Ventilation rates should be matched to conditions. If applicable, use process enclosures, local exhaust ventilation, or other engineering controls to maintain airborne levels below recommended exposure limits. If exposure limits have not been established, maintain airborne levels to an acceptable level. Eye wash facilities and emergency shower must be available when handling this product.
**Individual protection measures, such as personal protective equipment**	
**Eye/face protection**	Wear safety glasses with side shields (or goggles).
**Skin protection**	
**Hand protection**	Wear appropriate chemical resistant gloves.
**Other**	Wear appropriate chemical resistant clothing.
**Respiratory protection**	In case of insufficient ventilation, wear suitable respiratory equipment.
**Thermal hazards**	Wear appropriate thermal protective clothing, when necessary.
**General hygiene considerations**	Keep away from food and drink. Always observe good personal hygiene measures, such as washing after handling the material and before eating, drinking, and/or smoking. Routinely wash work clothing and protective equipment to remove contaminants.

9. Physical and chemical properties

**Appearance**	
**Physical state**	Liquid.
**Form**	Liquid.
**Color**	Colorless to Pale Yellow
**Odor**	Cheese
**Odor threshold**	Not available.
**pH**	Non-applicable.
**Melting point/freezing point**	Not available.
**Initial boiling point and boiling range**	Not available.
**Flash point**	> 200.0 °F (> 93.3 °C)
**Evaporation rate**	Not available.
**Flammability (solid, gas)**	Not applicable.
**Upper/lower flammability or explosive limits**	
**Flammability limit - lower (%)**	Not available.

**Flammability limit - upper (%)**	Not available.
**Explosive limit - lower (%)**	Not available.
**Explosive limit - upper (%)**	Not available.
**Vapor pressure**	Not available.
**Vapor density**	Not available.
**Relative density**	Not available.
**Solubility(ies)**	
**Solubility (water)**	Insoluble
**Partition coefficient (n-octanol/water)**	Not available.
**Auto-ignition temperature**	Not available.
**Decomposition temperature**	Not available.
**Viscosity**	Not available.
**Other information**	
**Explosive properties**	Not explosive.
**Oxidizing properties**	Not oxidizing.
**Specific gravity**	1.098 - 1.118 @25C
**VOC (Weight %)**	Not available.

10. Stability and reactivity

**Reactivity**	The product is stable and non-reactive under normal conditions of use, storage and transport.
**Chemical stability**	Material is stable under normal conditions.
**Possibility of hazardous reactions**	No dangerous reaction known under conditions of normal use.
**Conditions to avoid**	Avoid temperatures exceeding the flash point. Contact with incompatible materials.
**Incompatible materials**	Strong oxidizing agents.
**Hazardous decomposition products**	No hazardous decomposition products are known.

11. Toxicological information

Information on likely routes of exposure

**Inhalation**	Harmful if inhaled.
**Skin contact**	Harmful in contact with skin. Causes skin irritation.
**Eye contact**	Causes serious eye damage.
**Ingestion**	Harmful if swallowed.
**Symptoms related to the physical, chemical and toxicological characteristics**	Severe eye irritation. Symptoms may include stinging, tearing, redness, swelling, and blurred vision. Permanent eye damage including blindness could result. Skin irritation. May cause redness and pain.

Information on toxicological effects

**Acute toxicity**	Harmful if inhaled. Harmful in contact with skin. Harmful if swallowed.
**Skin corrosion/irritation**	Causes skin irritation.
**Serious eye damage/eye irritation**	Causes serious eye damage.
**Respiratory or skin sensitization**	
**Respiratory sensitization**	Not a respiratory sensitizer.
**Skin sensitization**	This product is not expected to cause skin sensitization.
**Germ cell mutagenicity**	No data available to indicate product or any components present at greater than 0.1% are mutagenic or genotoxic.
**Carcinogenicity**	This product is not considered to be a carcinogen by IARC, ACGIH, NTP, or OSHA.

OSHA Specifically Regulated Substances (29 CFR 1910.1001-1050)

Not listed.

**Reproductive toxicity**	This product is not expected to cause reproductive or developmental effects.

Specific target organ toxicity - single exposure Not classified.

Specific target organ toxicity - repeated exposure Not classified.

Aspiration hazard Not an aspiration hazard.

12. Ecological information

Ecotoxicity Very toxic to aquatic life. Toxic to aquatic life with long lasting effects.

Persistence and degradability No data is available on the degradability of this product.

Bioaccumulative potential

Partition coefficient n-octanol / water (log Kow)

BENZYL BENZOATE	3.97
BUTYRIC ACID	0.79
Hexanoic acid	1.88
PHENYLACETIC ACID	1.41

Mobility in soil No data available.

Other adverse effects No other adverse environmental effects (e.g. ozone depletion, photochemical ozone creation potential, endocrine disruption, global warming potential) are expected from this component.

13. Disposal considerations

Disposal instructions Collect and reclaim or dispose in sealed containers at licensed waste disposal site. Do not allow this material to drain into sewers/water supplies. Do not contaminate ponds, waterways or ditches with chemical or used container. Dispose of contents/container in accordance with local/regional/national/international regulations.

Local disposal regulations Dispose in accordance with all applicable regulations.

Hazardous waste code The waste code should be assigned in discussion between the user, the producer and the waste disposal company.

Waste from residues / unused products Dispose of in accordance with local regulations. Empty containers or liners may retain some product residues. This material and its container must be disposed of in a safe manner (see: Disposal instructions).

Contaminated packaging Since emptied containers may retain product residue, follow label warnings even after container is emptied. Empty containers should be taken to an approved waste handling site for recycling or disposal.

14. Transport information

SDS SECTION 14 DATA NOT VALID

DOT

Not regulated as dangerous goods.

IATA

Not regulated as dangerous goods.

IMDG

Not regulated as dangerous goods.

Transport in bulk according to Annex II of MARPOL 73/78 and the IBC Code Not established.

15. Regulatory information

US federal regulations This product is a "Hazardous Chemical" as defined by the OSHA Hazard Communication Standard, 29 CFR 1910.1200.

TSCA Section 12(b) Export Notification (40 CFR 707, Subpt. D)

Not regulated.

CERCLA Hazardous Substance List (40 CFR 302.4)

BUTYRIC ACID (CAS 107-92-6) Listed.

SARA 304 Emergency release notification

Not regulated.

OSHA Specifically Regulated Substances (29 CFR 1910.1001-1050)

Not listed.

Superfund Amendments and Reauthorization Act of 1986 (SARA)

Hazard categories
Immediate Hazard - Yes
Delayed Hazard - No
Fire Hazard - No
Pressure Hazard - No
Reactivity Hazard - No

SARA 302 Extremely hazardous substance

Not listed.

SARA 311/312 Hazardous chemical No

SARA 313 (TRI reporting)

Not regulated.

Other federal regulations

Clean Air Act (CAA) Section 112 Hazardous Air Pollutants (HAPs) List

Not regulated.

Clean Air Act (CAA) Section 112(r) Accidental Release Prevention (40 CFR 68.130)

Not regulated.

Safe Drinking Water Act (SDWA) Not regulated.

Drug Enforcement Administration (DEA). List 1 & 2 Exempt Chemical Mixtures (21 CFR 1310.12(c))

PHENYLACETIC ACID (CAS 103-82-2) 40 %WT

DEA Exempt Chemical Mixtures Code Number

PHENYLACETIC ACID (CAS 103-82-2) 8791

US state regulations

US. California Controlled Substances. CA Department of Justice (California Health and Safety Code Section 11100)

PHENYLACETIC ACID (CAS 103-82-2)

US. Massachusetts RTK - Substance List

BUTYRIC ACID (CAS 107-92-6)
Hexanoic acid (CAS 142-62-1)

US. New Jersey Worker and Community Right-to-Know Act

BUTYRIC ACID (CAS 107-92-6)
Hexanoic acid (CAS 142-62-1)

US. Pennsylvania Worker and Community Right-to-Know Law

BUTYRIC ACID (CAS 107-92-6)
Hexanoic acid (CAS 142-62-1)

US. Rhode Island RTK

BUTYRIC ACID (CAS 107-92-6)

US. California Proposition 65

California Safe Drinking Water and Toxic Enforcement Act of 1986 (Proposition 65): This material is not known to contain any chemicals currently listed as carcinogens or reproductive toxins.

16. Other information, including date of preparation or last revision

Issue date 04-08-2016

Revision date 04-11-2016

Version # 02

HMIS® ratings
Health: 3
Flammability: 0
Physical hazard: 0
Personal protection: B

Disclaimer To the best of our knowledge, the information provided in the Safety Data Sheet is accurate. It is intended to assist the user in his evaluation of the product's hazards, and safety precautions to be taken in its use. The data on this SDS relates only to the specific material designated herein. It is consistent with the state of general scientific and technical knowledge and should be used in accordance with professional guidelines. Arylessence, Inc. assumes no legal responsibility for use or reliance upon this data. See SDS for additional information.

Revision Information
Accidental release measures: Methods and materials for containment and cleaning up
Transport information: General information
