# Supplementary material for: Olfactory Stimuli Increase Presence in Virtual Environments
Source: PLoS One. 2016 Jun 16;11(6):e0157568. doi: 10.1371/journal.pone.0157568 (PMC4910977; doi:10.1371/journal.pone.0157568)
Supplement: S5 File — (PDF) [file pone.0157568.s005.PDF]

GARLIC TOY FRAGRANCE

Safety Data Sheet

according to Federal Register / Vol. 77, No. 58 / Monday, March 26, 2012 / Rules and Regulations

Date of issue: 05/30/2015

SECTION 1: Identification

1.1. Identification

Name : GARLIC TOY FRAGRANCE

1.2. Relevant identified uses of the substance or mixture and uses advised against

Use of the substance/mixture : Industrial uses: Uses of substances as such or in preparations* at industrial sites

1.3. Details of the supplier of the safety data sheet

Berje
700 Blair Road
Carteret, NJ 07008 - USA
T 973-748-8980 - F 973-680-9618
berje@berjeinc.com - www.berjeinc.com

1.4. Emergency telephone number

Emergency number : 1-800-424-9300 24 Hours Emergency Chemtrec. ERR - CCN2624

SECTION 2: Hazard(s) identification

2.1. Classification of the substance or mixture

GHS-US classification

Acute Tox. 4 (Oral)	H302 -	Harmful if swallowed
Aquatic Acute 2	H401 -	Toxic to aquatic life
Aquatic Chronic 2	H411 -	Toxic to aquatic life with long lasting effects

Full text of H-statements: see section 16

2.2. Label elements

GHS-US labelling

Hazard pictograms (GHS-US) :

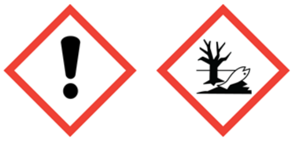

GHS07 GHS09

Signal word (GHS-US) : Warning

Hazard statements (GHS-US) : H302 - Harmful if swallowed
H401 - Toxic to aquatic life
H411 - Toxic to aquatic life with long lasting effects

Precautionary statements (GHS-US) : P264 - Wash hands thoroughly after handling
P270 - Do not eat, drink or smoke when using this product
P273 - Avoid release to the environment
P301+P312 - IF SWALLOWED: call a POISON CENTER or doctor/physician if you feel unwell
P330 - Rinse mouth
P391 - Collect spillage
P501 - Dispose in a safe manner in accordance with local/national regulations

2.3. Other hazards

No additional information available

2.4. Unknown acute toxicity (GHS US)

Not applicable

SECTION 3: Composition/information on ingredients

3.1. Substance

Not applicable

3.2. Mixture

Name	Product identifier	%	GHS-US classification
Trade Secret	Trade Secret	>= 80	Acute Tox. 4 (Oral), H302<br>Aquatic Acute 2, H401<br>Aquatic Chronic 2, H411

Full text of H-statements: see section 16

SECTION 4: First aid measures

4.1. Description of first aid measures

First-aid measures general : Never give anything by mouth to an unconscious person. If you feel unwell, seek medical advice (show the label where possible).

First-aid measures after inhalation : Allow breathing of fresh air. Allow the victim to rest.

First-aid measures after skin contact : Remove affected clothing and wash all exposed skin area with mild soap and water, followed by warm water rinse.

First-aid measures after eye contact : Rinse immediately with plenty of water. Obtain medical attention if pain, blinking or redness persist.

First-aid measures after ingestion : Rinse mouth. Do NOT induce vomiting. Obtain emergency medical attention. Call a POISON CENTER or doctor/physician if you feel unwell.

4.2. Most important symptoms and effects, both acute and delayed

Symptoms/injuries after ingestion : Swallowing a small quantity of this material will result in serious health hazard.

4.3. Indication of any immediate medical attention and special treatment needed

No additional information available

SECTION 5: Firefighting measures

5.1. Extinguishing media

Suitable extinguishing media : dry chemical powder. Foam. Dry powder. Carbon dioxide. Water spray. Sand.

Unsuitable extinguishing media : Do not use a heavy water stream.

5.2. Special hazards arising from the substance or mixture

No additional information available

5.3. Advice for firefighters

Firefighting instructions : Use water spray or fog for cooling exposed containers. Exercise caution when fighting any chemical fire. Prevent fire-fighting water from entering environment.

Protection during firefighting : Do not enter fire area without proper protective equipment, including respiratory protection.

SECTION 6: Accidental release measures

6.1. Personal precautions, protective equipment and emergency procedures

6.1.1. For non-emergency personnel

Emergency procedures : Evacuate unnecessary personnel.

6.1.2. For emergency responders

Protective equipment : Equip cleanup crew with proper protection.

Emergency procedures : Ventilate area.

6.2. Environmental precautions

Prevent entry to sewers and public waters. Notify authorities if liquid enters sewers or public waters. Avoid release to the environment.

6.3. Methods and material for containment and cleaning up

Methods for cleaning up : Soak up spills with inert solids, such as clay or diatomaceous earth as soon as possible. Collect spillage. Store away from other materials.

6.4. Reference to other sections

See Heading 8. Exposure controls and personal protection.

SECTION 7: Handling and storage

7.1. Precautions for safe handling

Precautions for safe handling : Wash hands and other exposed areas with mild soap and water before eating, drinking or smoking and when leaving work. Provide good ventilation in process area to prevent formation of vapour.

Hygiene measures : Do not eat, drink or smoke when using this product. Wash hands thoroughly after handling.

7.2. Conditions for safe storage, including any incompatibilities

Storage conditions	:	Keep only in the original container in a cool, well ventilated place away from : Keep container closed when not in use.
Incompatible products	:	Strong bases. Strong acids.
Incompatible materials	:	Sources of ignition. Direct sunlight.

SECTION 8: Exposure controls/personal protection

8.1. Control parameters

No additional information available

8.2. Exposure controls

Personal protective equipment	:	Avoid all unnecessary exposure.
Hand protection	:	Wear protective gloves/protective clothing/eye protection protective gloves.
Eye protection	:	Chemical goggles or safety glasses.
Respiratory protection	:	Wear appropriate mask.
Other information	:	Do not eat, drink or smoke during use.

SECTION 9: Physical and chemical properties

9.1. Information on basic physical and chemical properties

Physical state	:	Liquid
Colour	:	Colourless
Odour	:	characteristic
Odour threshold	:	No data available
pH	:	No data available
Melting point	:	No data available
Freezing point	:	No data available
Boiling point	:	No data available
Flash point	:	> 200 °F
Relative evaporation rate (butylacetate=1)	:	No data available
Flammability (solid, gas)	:	No data available
Explosive limits	:	No data available
Explosive properties	:	No data available
Oxidising properties	:	No data available
Vapour pressure	:	No data available
Relative density	:	1.118
Relative vapour density at 20 °C	:	No data available
Solubility	:	Water: Solubility in water of component(s) of the mixture :<br>• Benzyl benzoate: 153 mg/l (20 °C) • Allyl disulfide: 0.0071 g/100ml
Log Pow	:	No data available
Auto-ignition temperature	:	No data available
Decomposition temperature	:	No data available
Viscosity	:	No data available
Viscosity, kinematic	:	No data available
Viscosity, dynamic	:	No data available

9.2. Other information

No additional information available

SECTION 10: Stability and reactivity

10.1. Reactivity

No additional information available

10.2. Chemical stability

Not established.

10.3. Possibility of hazardous reactions

Not established.

10.4. Conditions to avoid

Direct sunlight. Extremely high or low temperatures.

10.5. Incompatible materials

Strong acids. Strong bases.

10.6. Hazardous decomposition products

fume. Carbon monoxide. Carbon dioxide.

SECTION 11: Toxicological information

11.1. Information on toxicological effects

Acute toxicity : Oral: Harmful if swallowed.

GARLIC TOY FRAGRANCE	
ATE US (oral)	1888.889 mg/kg bodyweight

Trade Secret	(*Chemical name, CAS number and/or exact concentration have been withheld as a trade secret)
LD50 oral rat	1870 mg/kg (Rat; OECD 401: Acute Oral Toxicity; Experimental value; >2000 mg/kg bodyweight; Rat)
LD50 dermal rat	4400 mg/kg (Rat)
LD50 dermal rabbit	4000 mg/kg (Rabbit; Experimental value; Modification of Draize 1959 method; >2; Rabbit)
ATE US (oral)	1870.000 mg/kg bodyweight
ATE US (dermal)	4000.000 mg/kg bodyweight

Skin corrosion/irritation : Not classified

Serious eye damage/irritation : Not classified

Respiratory or skin sensitisation : Not classified

Germ cell mutagenicity : Not classified
Based on available data, the classification criteria are not met

Carcinogenicity : Not classified

Reproductive toxicity : Not classified
Based on available data, the classification criteria are not met

Specific target organ toxicity (single exposure) : Not classified

Specific target organ toxicity (repeated exposure) : Not classified

Aspiration hazard : Not classified

Potential adverse human health effects and symptoms : Harmful if swallowed.

Symptoms/injuries after ingestion : Swallowing a small quantity of this material will result in serious health hazard.

SECTION 12: Ecological information

12.1. Toxicity

Ecology - water : Toxic to aquatic life with long lasting effects.

Trade Secret	(*Chemical name, CAS number and/or exact concentration have been withheld as a trade secret)
LC50 fish 1	4.6 mg/l (96 h; Pisces; GLP)
EC50 Daphnia 1	426 mg/l (24 h; Daphnia magna; Locomotor effect)
LC50 fish 2	2.32 mg/l (96 h; Brachydanio rerio)
EC50 Daphnia 2	309 mg/l (48 h; Daphnia magna; Locomotor effect)
Threshold limit algae 1	647 mg/l (72 h; Pseudokirchneriella subcapitata; Biomass)
Threshold limit algae 2	247 mg/l (72 h; Pseudokirchneriella subcapitata; Growth rate)

12.2. Persistence and degradability

GARLIC TOY FRAGRANCE	
Persistence and degradability	May cause long-term adverse effects in the environment.

Trade Secret	(*Chemical name, CAS number and/or exact concentration have been withheld as a trade secret)
Persistence and degradability	Readily biodegradable in water. Low potential for mobility in soil.

12.3. Bioaccumulative potential

GARLIC TOY FRAGRANCE	
Bioaccumulative potential	Not established.

Trade Secret	(*Chemical name, CAS number and/or exact concentration have been withheld as a trade secret)
BCF fish 1	2286 (Pisces)
Log Pow	3.88 - 4
Bioaccumulative potential	Low potential for bioaccumulation (Log Kow < 4).

12.4. Mobility in soil

Trade Secret	(*Chemical name, CAS number and/or exact concentration have been withheld as a trade secret)
Surface tension	0.027 N/m (210 °C)

12.5. Other adverse effects

Effect on the global warming : No known ecological damage caused by this product.

Other information : Avoid release to the environment.

SECTION 13: Disposal considerations

13.1. Waste treatment methods

Waste disposal recommendations : Dispose in a safe manner in accordance with local/national regulations. Dispose in a safe manner in accordance with local/national regulations.

Ecology - waste materials : Avoid release to the environment.

SECTION 14: Transport information

Department of Transportation (DOT)

In accordance with DOT

Not regulated for transport

TDG

No additional information available

Transport by sea

UN-No. (IMDG) : 3082

Proper Shipping Name (IMDG) : ENVIRONMENTALLY HAZARDOUS SUBSTANCE, LIQUID, N.O.S.

Class (IMDG) : 9 - Miscellaneous dangerous substances and articles

Packing group (IMDG) : III - substances presenting low danger

Air transport

UN-No. (IATA) : 3082

Proper Shipping Name (IATA) : Environmentally hazardous substance, liquid, n.o.s.

Class (IATA) : 9 - Miscellaneous Dangerous Goods

Packing group (IATA) : III - Minor Danger

SECTION 15: Regulatory information

15.1. US Federal regulations

Trade Secret	(*Chemical name, CAS number and/or exact concentration have been withheld as a trade secret)
Listed on the United States TSCA (Toxic Substances Control Act) inventory	

15.2. International regulations

CANADA

No additional information available

EU-Regulations
No additional information available

National regulations
No additional information available

15.3. US State regulations
No additional information available

SECTION 16: Other information

Data sources	:	REGULATION (EC) No 1272/2008 OF THE EUROPEAN PARLIAMENT AND OF THE COUNCIL of 16 December 2008 on classification, labelling and packaging of substances and mixtures, amending and repealing Directives 67/548/EEC and 1999/45/EC, and amending Regulation (EC) No 1907/2006.
Other information	:	None.

Full text of H-statements:

Acute Tox. 4 (Oral)	Acute toxicity (oral), Category 4
Aquatic Acute 2	Hazardous to the aquatic environment — Acute Hazard, Category 2
Aquatic Chronic 2	Hazardous to the aquatic environment — Chronic Hazard, Category 2
H302	Harmful if swallowed
H401	Toxic to aquatic life
H411	Toxic to aquatic life with long lasting effects

HMIS III Rating

Health	:	1 Slight Hazard - Irritation or minor reversible injury possible
Flammability	:	1 Slight Hazard - Materials that must be preheated before ignition will occur. Includes liquids, solids and semi solids having a flash point above 200 F. (Class IIIB)
Physical	:	0 Minimal Hazard - Materials that are normally stable, even under fire conditions, and will NOT react with water, polymerize, decompose, condense, or self-react. Non-Explosives.
Personal Protection	:	B<br>B - Safety glasses, Gloves

SDS US Trade Secret (Berje) 5.2

This information is based on our current knowledge and is intended to describe the product for the purposes of health, safety and environmental requirements only. It should not therefore be construed as guaranteeing any specific property of the product
